# Modelling of Anisotropic Electrical Conduction in Layered Structures 3D-Printed with Fused Deposition Modelling

**DOI:** 10.3390/s21113710

**Published:** 2021-05-26

**Authors:** Alexander Dijkshoorn, Martijn Schouten, Stefano Stramigioli, Gijs Krijnen

**Affiliations:** 1 Robotics and Mechatronics Group (RAM), University of Twente, 7500 AE Enschede, The Netherlands; m.schouten@utwente.nl (M.S.); s.stramigioli@utwente.nl (S.S.); gijs.krijnen@utwente.nl (G.K.); 2 Biomechatronics and Energy-Efficient Robotics Lab, ITMO University, 197101 Saint Petersburg, Russia

**Keywords:** 3D printing, electrical resistivity, anisotropy, fused deposition modeling, track-elements, 3D-printed sensors

## Abstract

3D-printing conductive structures have recently been receiving increased attention, especially in the field of 3D-printed sensors. However, the printing processes introduce anisotropic electrical properties due to the infill and bonding conditions. Insights into the electrical conduction that results from the anisotropic electrical properties are currently limited. Therefore, this research focuses on analytically modeling the electrical conduction. The electrical properties are described as an electrical network with bulk and contact properties in and between neighbouring printed track elements or traxels. The model studies both meandering and open-ended traxels through the application of the corresponding boundary conditions. The model equations are solved as an eigenvalue problem, yielding the voltage, current density, and power dissipation density for every position in every traxel. A simplified analytical example and Finite Element Method simulations verify the model, which depict good correspondence. The main errors found are due to the limitations of the model with regards to 2D-conduction in traxels and neglecting the resistance of meandering ends. Three dimensionless numbers are introduced for the verification and analysis: the anisotropy ratio, the aspect ratio, and the number of traxels. Conductive behavior between completely isotropic and completely anisotropic can be modeled, depending on the dimensionless properties. Furthermore, this model can be used to explain the properties of certain 3D-printed sensor structures, like constriction-resistive strain sensors.

## 1. Introduction

3D-printing objects with embedded conductive structures for sensing by means of Fused Deposition Modeling (FDM) is an upcoming field of research [1,2], which is particularly popular for electrochemical [3,4] and electromechanical sensors [2,5]. Notable examples include elastic, piezoresistive strain sensors [6,7], temperature sensors [8], structural vibration sensors [9,10], and soft EMG sensors [11], among others. Additionally, the FDM-printing of conductive structures is used for a wide field of applications outside of sensing, such as electromagnetic interference shielding [12,13], electrical heating [14,15], electrical circuits [8,16,17], soft robotics [18], and electrochemical energy storage [19,20].

**Figure 1:**
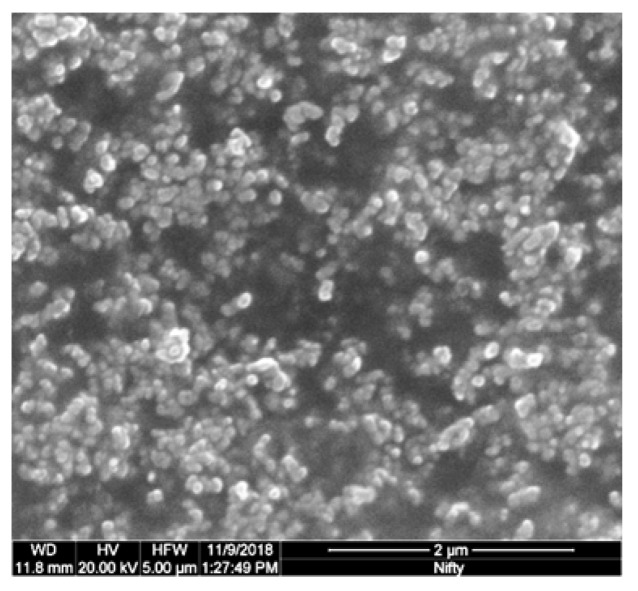
Scanning Electron Microscopy (SEM) image of the Carbon Black (CB) distribution in unprinted filament, showing CB particles and aggregates. The sample is prepared by the cryo-fracturing of PI-eTPU 85-700+ filament and imaged with an FEI Quanta 450.

The 3D-printing technique by FDM is a widely used 3D-printing technique, which builds up objects by heating and extruding thermoplastics, after which the plastic is deposited traxel-by-traxel (i.e., track-element) and layer-by-layer on a build platform, where it then solidifies. FDM is an interesting 3D-printing method for sensors because of its multi-material ability and shape-freedom, using conductive and non-conductive filaments [21,22]. The conductive filaments are mostly made from thermoplastics that are filled with conductive nano-particles, called conductive polymer composites (CPC). A significant amount of research has been done on the electrical properties of conductive-polymer composites for 3D-printing [16,17,23]. The most common CPC’s use carbon-based conductive particles (carbon black, carbon nano-tubes, and graphene) [17], since these materials are low-cost, readily available, and chemically stable [24]. The electrical properties of CPC’s are caused by their conductive networks [25], where, above a certain conductive filler concentration (the percolation threshold), a conductive network is formed [26,27]. The resistivity in CPC’s is mainly accounted for by the quantum tunnelling between local conductive networks and particle aggregates [25,26]. In Figure 1, carbon black particles and aggregates can be recognized in a commercially available conductive filament (PI-eTPU 85-700+ [28]).

**Figure 2:**
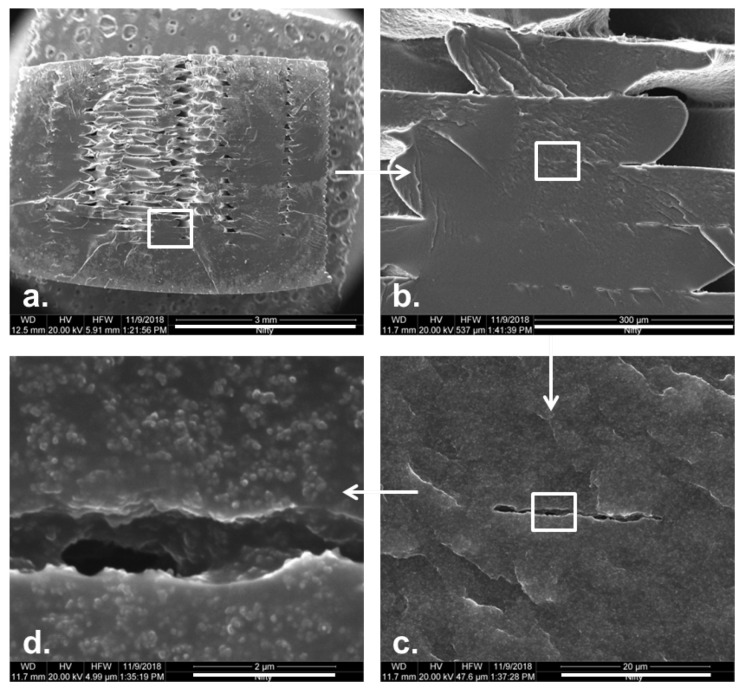
The SEM images of a cross section of a 3D-printed sample with cross-ply infill, prepared with cryo-fracturing: (**a**). the full cross section (scale bar 3 mm), (**b**). several traxels (scale bar 300 μm), (**c**). void between traxels (scale bar 20 μm), and (**d**). void close-up showing carbon black particles in the void (scale bar 2 μm).

Besides the material properties, the FDM printing process itself also influences the conductive properties of 3D-prints. The layerwise printing process creates a meso-structure with anisotropic physical properties, which gives rise to metamaterials. The anisotropic properties have been studied for the mechanical [29,30,31], thermal [32,33], magnetic [34], and electrical [35] domains. The anisotropy in the electrical properties can be explained by means of interfacial and inter-layer contact resistance, which is believed to originate from imperfect bonding conditions (e.g. the presence of voids) [35,36,37,38] and the inhomogeneous distribution of the conductive particles (especially in between traxels and layers) [27,39,40,41]. For low numbers of layers, the thickness variation is also of importance, as Gnanasekaran et al. showed for a varying layer thickness with a factor of 1.38 due to ridges [27]. It is important to note that the electrical anisotropy can be measured both in-plane and out-of-plane [27,35,41], where the inter-layer properties also give rise to capacitive properties [40]. Figure 2 shows a cross section of a 3D-printed rectangular bar with cross-ply infill with voids between the layers, with gap sizes in the order of 1 μm. Carbon black particles can be observed inside the gap. Each subimage represents a zoomed-in version in the white box.

Modelling and characterization methods for studying anisotropic electrical conduction are required to understand and predict the performance of 3D-printed sensors [42] because the electrical anisotropic properties also influence the performance of 3D-printed sensors [36,41].

Only a few models of the anisotropic electrical conduction in 3D-prints can be found in the literature, where a concise overview is presented in [2]. The anisotropic conduction can be modeled by defining a different traxel, inter-traxel, and inter-layer resistance, which was demonstrated with a 1D example presented by Hampel et al. [36]. The directional resistivity components can be described with homogenized values, as shown for 3D-prints [29] and carbon fiber reinforced polymers (CFRP) [43]. Fields of study regarding electrical anisotropic materials outside of 3D-printing utilize electrical networks for modeling, such as conductive fabrics [44] and CFRP [45]. This has also been used by Truman et al. to study the effect of local resistance variations in resistor networks to represent 3D printed conductors [13]. Finally, FEM simulations are used to study anisotropy in all directions by taking a unit cell to represent a piece of material with voids and layer bonding [35]. Because voids, raster orientation, and bonding are important for the anisotropic conductivity [41], these features need to be included in the modeling. In previous work, the authors also used FEM simulations based on the model of Hampel et al. to simulate the in-plane anisotropy by including the inter-traxel resistance and meanders [42]. However, homogenized models cannot include local features, electrical network models do not present interpretable results with high resolution and simplified (1D) analytical models, and numerical and FEM simulations lack insightful information regarding anisotropic conduction. An analytical model that takes inter-traxel resistance and geometry (e.g., meanders) into account is expected to provide this kind of information.

Therefore, the goal of this study is to explore the effect of the anisotropic electrical properties on the electrical conduction in 3D-prints by means of deriving and studying a 2D analytical model as well as deriving some determining factors. Such a model can also provide faster calculations than a full FEM simulation, for example, in parametric studies. The model analyzes the voltage and current distributions, the effects of the inter-traxel resistance, the meandering connections of traxels, and the effect of the aspect ratio of samples. It does so by taking bulk and contact (inter-traxel or inter-layer) resistance into account, in order to represent the phenomena caused by the anisotropic meso-structure.

The outline of the paper is as follows. First, the phenomenological 2D model is derived and analyzed. Afterwards, the methods for validation and verification are outlined. In the Section 4, the model verification is presented and the model is used to analyze the effect of inter-traxel resistance, aspect ratio, number of traxels, and meanders. Finally, the model is used to analyze a type of 3D-printed sensor and the results are discussed.

This research presents a comprehensive analytical model, which gives insight into the anisotropic conduction in 3D-prints. In such a model, depending on the anisotropy ratio, aspect ratio, and number of traxels, conduction can range from isotropically spreading through the bulk to anisotropically following the printed traxels.

## 2. Theoretical Model

**Figure 3:**
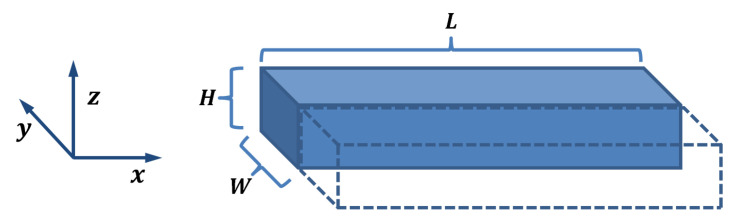
The schematical drawing of the 3D-printed traxels present in the model, with length *L*, width *W*, and height *H*. The cross section is drawn as a rectangular shape to clearly indicate the main dimensions; however, the actual cross section looks more like a trapezoid with rounded corners, like in Figure 2b.

**Figure 4:**
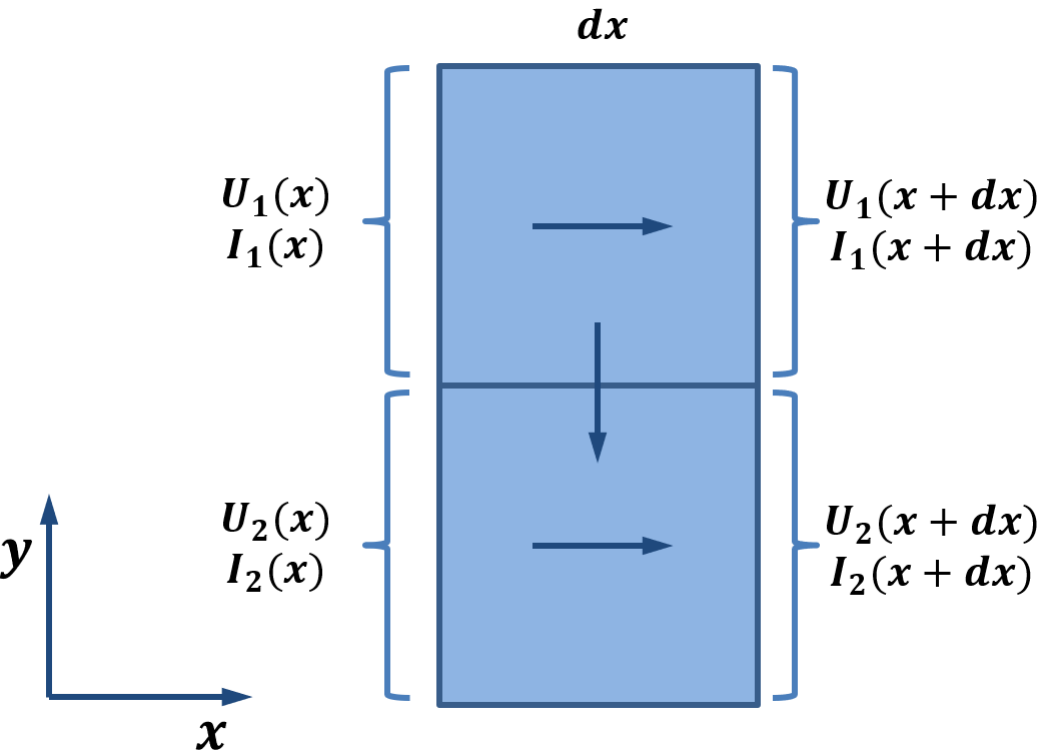
Schematic interaction of the voltage and current between two neighbouring traxels slices of Δx wide.

The electrical conduction in 3D-printed structures is modeled as a collection of traxels, which represent the discrete deposited line elements, of which an FDM 3D-printed material consists. Figure 3 shows the geometrical lay-out of a print, where a modeled traxel has a length of *L* in *x*-direction, is *W* wide in *y*-direction, and is *H* high in the *z*-direction. In this research, a 2D-model is studied, which represents a single horizontal layer (a 2D cross section of multiple layers would also be possible). Hence, traxels are stacked against each other in *y*-direction and voltage, and the current boundary conditions are prescribed on the ends of the track (the yz-planes). The anisotropic properties with the inter-traxel and inter-layer properties that are discussed in the previous section are represented through bulk properties within a traxel (i.e., intra-traxel properties) and contact properties between traxels (i.e., inter-traxel properties). Every traxel has a certain bulk resistivity and permittivity, whereas the traxels are connected via contact resistance and contact capacitance over their contact surfaces. Because of these electrical connections, current can flow through traxels and to neighbouring traxels, as shown in Figure 4. This interaction leads to changing potentials and currents in the individual traxels, which depend on each other and need to be solved together. For *N* traxels, there are 2N equations to be solved. The potential and current relations lead to a set of coupled second order differential equations that are solved using an eigenmode/eigenvector solution with coefficients being determined by the (given) boundary conditions.

**Figure 5:**
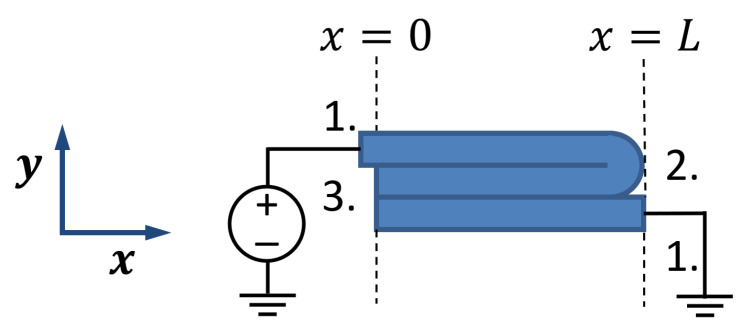
The possible boundary conditions for traxels: 1. Prescribed Voltage, 2. Meandering End, and 3. Open End.

There are three possible boundary conditions for every traxel, as illustrated in Figure 5. They all have their own implications for the voltage U(x,t) and current I(x,t) at the boundaries x=0 and x=L:

1. Prescribed Voltage: a fixed voltage at a boundary because of the presence of a terminal or ground.
  (1) $$U_{n}\left(0,t\right)=U_{\mathrm{prescribed}}$$
2. Meandering End: a connection to another traxel (normally a neighbouring traxel), causing an equal voltage and an equal but opposite current.
  (2) $$U_{n}\left(L,t\right)=U_{n-1}\left(L,t\right)$$
  (3) $$I_{n}\left(L,t\right)=-I_{n-1}\left(L,t\right)$$
3. Open End: a floating electrical voltage and no current flowing, since there is no end connection with other traxels or sources. This could represent stacked layers or traxels without meandering ends.
  (4) $$I_{n}\left(0,t\right)\propto \frac{\partial U_{n}\left(0,t\right)}{\partial x}=0$$

**Figure 6:**
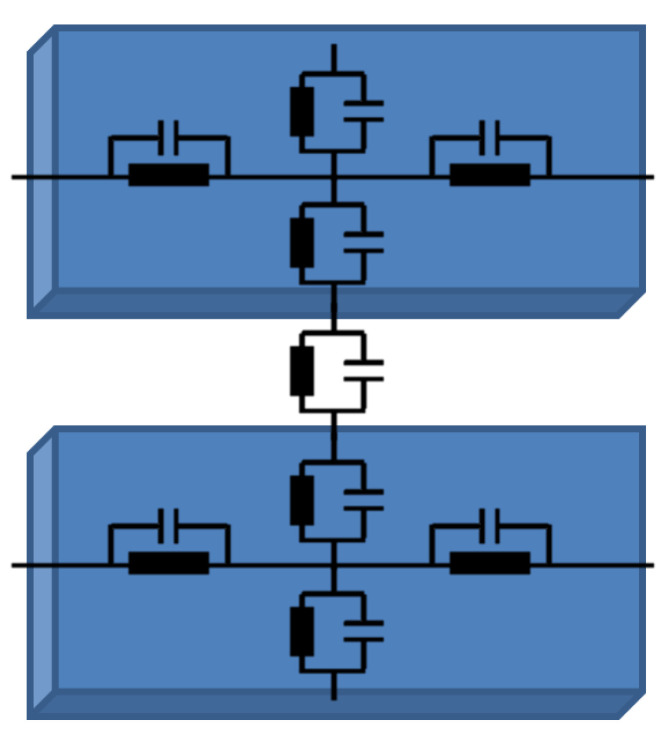
An equivalent circuit representation for the electrical properties of a slice of traxel.

The mathematical model can be derived by considering the distributed electrical properties of the traxels and the contacts between the traxels, being represented by an equivalent network, as shown in Figure 6, and as we used before for the FEM simulations shown in [42].

### 2.1. Model Derivation

The model derivation starts from the expressions of the bulk and contact properties for a piece of traxel of Δx wide, such as the slice presented in Figure 3. The bulk resistance can be defined as $R_{\mathrm{bulk}}=\frac{\rho \Delta x}{HW}$, with ρ being the volume resistivity of the material shown in Ωm. The bulk capacitance arises due to the permittivity of the material, and it is described with $C_{\mathrm{bulk}}=\frac{\epsilon_{0}\epsilon_{r}HW}{\Delta x}$ with $\epsilon_{0}$ being the permittivity of vacuum in $F/m^{-1}$ and with $\epsilon_{r}$ as the relative permittivity. The contact resistance is defined as $R_{\mathrm{contact}}=\frac{\sigma }{A}=\frac{\sigma }{H\Delta x}$, with σ being the contact resistivity between two tracks in $\Omega m^{2}$. Finally, the contact capacitance can be expressed as $C_{\mathrm{contact}}=C_{0}H\Delta x$, with $C_{0}$ being the contact capacitance per contact area in $F/m^{-2}$.

Parallel traxels are placed in-plane, directly next to each other in the model. Each traxel has four important variables. On each end, a current or voltage condition is present. The system is solved in terms of the potential, where the boundary conditions are given in either $U=U_{\mathrm{in}/\mathrm{out}}$ or $\frac{\partial U}{\partial x}=0$. For every traxel, this yields two variables, the voltage U(x,t) and its spatial derivative. The model aims at solving these two variables at every location *x*. Based on the bulk and contact properties shown in Figure 6, separate equations can be derived and put together. This method presents a quasi-analytical model with lumped traxels, which is quite similar to the models from the Finite Difference Method. In the following sections, the intra-traxel and inter-traxel relations for the current and voltage are derived and combined, which yields the system of equations. The system of equations is solved and the different types of boundary conditions are elaborated on.

#### 2.1.1. Bulk Properties

**Figure 7:**
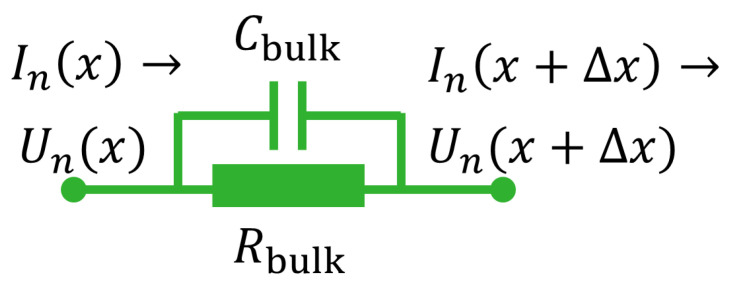
Equivalent circuit representation for the horizontal electrical bulk properties of a slice of traxel Δx wide.

A single piece of traxel is represented by means of a parallel resistor and capacitor, as schematically shown in Figure 7. The piece of traxel is Δx long, has potentials $U_{n}\left(x\right)$ and $U_{n}\left(x+\Delta x\right)$ on its ends, and a current $I_{n}$ flows through. For the equations, an arbitrary nth traxel is considered red, therefore indicating the variables *U* and *I* with subscript *n*. The bulk resistance can then be expressed with $R_{\mathrm{bulk}}=\frac{\rho \Delta x}{HW}$ and the bulk capacitance with $C_{\mathrm{bulk}}=\frac{\epsilon_{0}\epsilon_{r}HW}{\Delta x}$. The current flows through the traxels from left to right. On the left, the current of traxel *n* can be expressed to be the current that flows due to the potential difference over the section:

(5) $$I_{n}\left(x,t\right)=\frac{\Delta U_{n}\left(x,t\right)}{R_{\mathrm{bulk}}}+C_{\mathrm{bulk}}\frac{\partial (\Delta U_{n}\left(x,t\right))}{\partial t}$$

By substituting the expressions for $R_{\mathrm{bulk}}$ and $C_{\mathrm{bulk}}$, one obtains:

(6) $$I_{n}\left(x,t\right)=-\frac{U_{n}\left(x+\Delta x,t\right)-U_{n}\left(x,t\right)}{\frac{\rho }{HW}\Delta x}-\frac{\epsilon_{0}\epsilon_{r}HW}{\Delta x}\frac{\partial }{\partial t}\left(U_{n}\left(x+\Delta x,t\right)-U_{n}\left(x,t\right)\right)$$

When taking the slice of traxel infinitesimally thin, the expression can be rewritten with partial derivatives:

(7) $$I_{n}\left(x,t\right)=-HW\left(\frac{1}{\rho }\frac{\partial U_{n}\left(x,t\right)}{\partial x}+\epsilon_{0}\epsilon_{r}\frac{\partial }{\partial t}\frac{\partial U_{n}\left(x,t\right)}{\partial x}\right)$$

Assuming only harmonic expressions for $U_{n}$ and $I_{n}$, the Fourier transform can be applied, which results in:

(8) $${\hat{I}}_{n}\left(x,\omega \right)=-HW\left(\frac{1}{\rho }+j\omega \epsilon_{0}\epsilon_{r}\right)\frac{\partial {\hat{U}}_{n}\left(x,\omega \right)}{\partial x}$$

where the $\hat{\;}$ indicates the complex Fourier amplitudes. This expression can be differentiated with respect to *x* to give a second order expression in ${\hat{U}}_{n}$:

(9) $$\frac{\partial {\hat{I}}_{n}\left(x,\omega \right)}{\partial x}=-HW\left(\frac{1}{\rho }+j\omega \epsilon_{0}\epsilon_{r}\right)\frac{\partial^{2}{\hat{U}}_{n}\left(x,\omega \right)}{\partial x^{2}}$$

#### 2.1.2. Contact Properties

**Figure 8:**
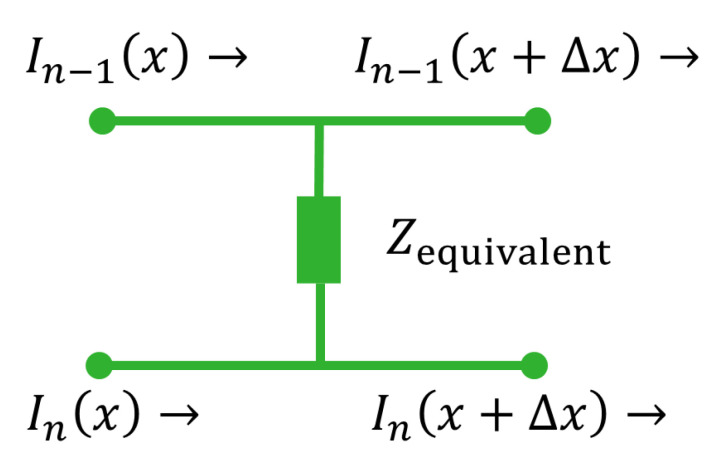
The equivalent impedance circuit representation for the electrical contact properties combined with the vertical bulk properties of a slice of Δx wide of two neighbouring traxels.

The contact between two pieces of traxel is also represented by means of a resistor and capacitor in parallel. Representing the vertical bulk properties (the voltage drops perpendicular to the contacts) in this approach is one challenge of this approach. This can be solved by lumping the vertical traxel properties and adding it in series as additional term in the contact properties, based on the equivalent network in Figure 6 to represent half of the traxel on both sides of the contact. An equivalent impedance like in Figure 8 can be derived for this circuit, for which the derivation is given in Appendix A:

(10) $${\hat{Z}}_{\mathrm{eq}}\left(\omega \right)=\frac{1}{H\Delta x}\;\cdot \;\frac{\rho W+\sigma +j\omega \rho \sigma WC_{0}+j\omega \rho \sigma \epsilon_{0}\epsilon_{r}}{1+j\omega \rho \epsilon_{0}\epsilon_{r}+j\omega \sigma C_{0}-\omega^{2}\rho \sigma \epsilon_{0}\epsilon_{r}C_{0}}$$

**Figure A1:**
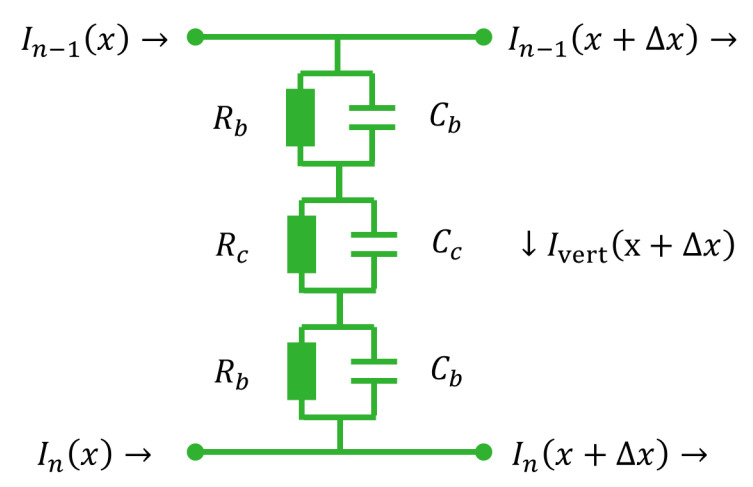
Equivalent circuit representation for the electrical contact properties *c* combined with the vertical bulk properties *b* of a slice of two neighbouring traxels of Δx wide.

The pieces of traxel shown in Figure A1 are Δx long. For the equations, an arbitrary nth traxel is considered, with a neighbouring traxel n−1. The current flows through the traxels from left to right and can also flow through the contact impedance due to the potential difference. The equivalent impedance is already expressed in the Fourier transformed form, while assuming harmonic functions. This is also done for the current balance:

(11) $$\left({\hat{I}}_{n-1}\left(x+\Delta x,\omega \right)-{\hat{I}}_{n-1}\left(x,\omega \right)\right)=\frac{{\hat{U}}_{n}\left(x,\omega \right)-{\hat{U}}_{n-1}\left(x,\omega \right)}{{\hat{Z}}_{\mathrm{eq}}\left(\omega \right)}$$

Furthermore, a dimensionless parameter Γ is introduced (which gives the ratio between the bulk and contact properties from Equations (9)–(11):

(12) $$\Gamma \left(\omega \right)=\frac{1}{\frac{1}{\rho }+j\omega \epsilon_{0}\epsilon_{r}}\;\cdot \;\frac{W}{H\Delta x}\;\cdot \;\frac{1}{{\hat{Z}}_{\mathrm{eq}}}$$

By rewriting, we find:

(13) $$\Gamma =\frac{\left(\frac{\rho }{1+j\omega \rho \epsilon_{0}\epsilon_{r}}\right)}{\left(\frac{\rho }{1+j\omega \rho \epsilon_{0}\epsilon_{r}}\right)+\left(\frac{\sigma /W}{1+j\omega \sigma C_{0}}\right)}$$

which is possible, since these are all physical parameters, and it holds that: $1+j\omega \rho \epsilon_{0}\epsilon_{r}\neq 0$ and, hence, there are no singularities upon division. From Equation (13), it becomes clear that Γ represents the horizontal impedivity terms (purely bulk properties) over the vertical impedivity terms (bulk and contact properties). This indicates that it always holds that $\left|\Gamma \right|\leq 1$, since, in practice, $\rho ,\sigma ,\epsilon_{0},\epsilon_{r},C_{0},\omega$ are all real and positive values.

Substituting the expression for Γ from Equation (12) and the equivalent impedance from Equation (10) into the expression for the contact properties, Equation (11) yields:

(14) $$\left({\hat{I}}_{n-1}\left(x+\Delta x,\omega \right)-{\hat{I}}_{n-1}\left(x,\omega \right)\right)=\frac{H\Delta x}{W}\left(\frac{1}{\rho }+j\omega \epsilon_{0}\epsilon_{r}\right)\Gamma \left({\hat{U}}_{n}\left(x,\omega \right)-{\hat{U}}_{n-1}\left(x,\omega \right)\right)$$

When taking the slice of traxels infinitesimally thin, the differences in the expression can be rewritten as partial derivatives:

(15) $$\frac{\partial {\hat{I}}_{n-1}\left(x,\omega \right)}{\partial x}=\frac{H}{W}\left(\frac{1}{\rho }+j\omega \epsilon_{0}\epsilon_{r}\right)\Gamma \left({\hat{U}}_{n}\left(x,\omega \right)-{\hat{U}}_{n-1}\left(x,\omega \right)\right)$$

#### 2.1.3. Combined Properties

The full system of equations can be found by combining the equations for bulk and contact properties by substituting Equation (15) into Equation (9) and rewriting (the term $-H(\frac{1}{\rho }+j\omega \epsilon_{0}\epsilon_{r})$ occurs on both sides of the equation and drops out):

(16) $$\frac{\partial^{2}{\hat{U}}_{n-1}\left(x,\omega \right)}{\partial x^{2}}+\Gamma \left(\omega \right)\frac{\left\{{\hat{U}}_{n}\left(x,\omega \right)-{\hat{U}}_{n-1}\left(x,\omega \right)\right\}}{W^{2}}=0$$

Equation (16) describes the case for a traxel with a single neighbour, hence being at the edge of a printed sample. However, for traxels not at the edge, there is a neighbouring traxel on both sides, and an additional term for the second neighbouring traxel needs to be included.

(17) $$\frac{\partial^{2}{\hat{U}}_{n}\left(x,\omega \right)}{\partial x^{2}}+\Gamma \left(\omega \right)\frac{\left\{-{\hat{U}}_{n-1}\left(x,\omega \right)+2{\hat{U}}_{n}\left(x,\omega \right)-{\hat{U}}_{n+1}\left(x,\omega \right)\right\}}{W^{2}}=0$$

Equation (17) needs to be solved for every traxel with two neighbours, whereas Equation (16) needs to be solved for the two traxels on the outer edge. Accordingly, in this way, any 2D structure with a finite number of parallel traxels can be described by this system of coupled equations.

#### 2.1.4. Solving the Equations

**Figure 9:**
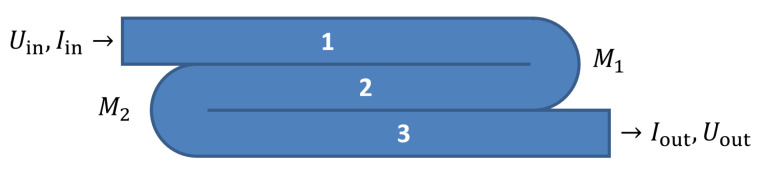
A schematic image of an example with three traxels, indicating the layout for the implicit boundary condition example.

Equations (16) and (17) can be combined into a system of equations that describes the entire system. The system can be turned into an eigenvalue problem to solve this system of equations. This is illustrated with an example involving three traxels, from which the traxels are numbered from 1 to 3, as shown in Figure 9. The system consists of three second order differential equations, hence the expected outcomes are something with six eigenvalues:

(18) $${\hat{U}}_{n}\left(x,\omega \right)=\sum_{i=1}^{6}C_{i}e^{\lambda_{i}x}$$

Substituting this in the system equations for the second row (n=2) yields, e.g.,:

(19) $$\lambda^{2}{\hat{U}}_{2}\left(x,\omega \right)+\Gamma \left(\omega \right)\frac{\left\{-{\hat{U}}_{1}\left(x,\omega \right)+2{\hat{U}}_{2}\left(x,\omega \right)-{\hat{U}}_{3}\left(x,\omega \right)\right\}}{W^{2}}=0$$

Combining the three equations in matrix form yields the eigenvalue problem ($\left(A-\lambda^{2}I\right)\vec{U}=\vec{0}$), where, in this specific case, the eigenvalues are given by $\lambda^{2}$ and the whole thing is multiplied by −1:

(20) $$\left[\begin{array}{ccc} \left(\lambda^{2}+\frac{\Gamma }{W^{2}}\right) & -\frac{\Gamma }{W^{2}} & 0\\ -\frac{\Gamma }{W^{2}} & \left(\lambda^{2}+2\frac{\Gamma }{W^{2}}\right) & -\frac{\Gamma }{W^{2}}\\ 0 & -\frac{\Gamma }{W^{2}} & \left(\lambda^{2}+\frac{\Gamma }{W^{2}}\right) \end{array}\right]\left[\begin{array}{c} U_{1}\\ U_{2}\\ U_{3} \end{array}\right]=\left[\begin{array}{c} 0\\ 0\\ 0 \end{array}\right]$$

The eigenvalue problem has a nontrivial solution when the determinant of the matrix equals 0. From this, the eigenvalues are found to be $\lambda^{2}=0$, $\lambda^{2}=\frac{\Gamma }{W^{2}}$ and $\lambda^{2}=3\frac{\Gamma }{W^{2}}$. In this way, six individual terms for λ and the corresponding eigenvectors are obtained:

(21) $$\begin{array}{ccc} \; & \lambda_{1}=-\lambda_{2}=0, & \;\;\;{\vec{\nu }}_{1}={\vec{\nu }}_{2}={\left(1,1,1\right)}^{T} \end{array}$$

(22) $$\begin{array}{ccc} \; & \lambda_{3}=-\lambda_{4}=-\sqrt{\frac{\Gamma }{W^{2}}}, & \;\;\;{\vec{\nu }}_{3}={\vec{\nu }}_{4}={\left(-1,0,1\right)}^{T} \end{array}$$

(23) $$\begin{array}{ccc} \; & \lambda_{5}=-\lambda_{6}=-\sqrt{3\frac{\Gamma }{W^{2}}}, & \;\;\;{\vec{\nu }}_{5}={\vec{\nu }}_{6}={\left(1,-2,1\right)}^{T} \end{array}$$

The solutions that arise from these eigenvalues and vectors are in the form of (with $\vec{\hat{U}}\left(x,\omega \right)$ indicating that it is a vector with the complex voltage amplitude for every traxel):

(24) $$\vec{\hat{U}}\left(x,\omega \right)=\alpha_{1}{\vec{\nu }}_{1}+\alpha_{2}{\vec{\nu }}_{2}x+\alpha_{3}{\vec{\nu }}_{3}e^{\lambda_{3}x}+\alpha_{4}{\vec{\nu }}_{4}e^{\lambda_{4}x}+\alpha_{5}{\vec{\nu }}_{5}e^{\lambda_{5}x}+\alpha_{6}{\vec{\nu }}_{6}e^{\lambda_{6}x}$$

### 2.2. Boundary Conditions

Equation (24) shows that, for three (N) layers, we find six (2N) eigenvalues, six (2N) eigenvectors, and six (2N) unknown $\alpha_{i}$s. This means that we need six (2N) boundary conditions to solve all $\alpha_{i}$s. By using boundary conditions on both sides of each traxel, for either voltage or current, the coefficients $\alpha_{i}$ can be found. For the situations where the current is taken as boundary, the derivative of Equation (24) has to be used (since ${\hat{I}}_{n}\left(x,\omega \right)\propto \frac{\partial {\hat{U}}_{n}\left(x,\omega \right)}{\partial x}$):

(25) $$\frac{\partial \vec{\hat{U}}\left(x,\omega \right)}{\partial x}=\alpha_{2}{\vec{\nu }}_{1}+\lambda_{3}\alpha_{3}{\vec{\nu }}_{2}e^{\lambda_{3}x}+\lambda_{4}\alpha_{4}{\vec{\nu }}_{2}e^{\lambda_{4}x}+\lambda_{5}\alpha_{5}{\vec{\nu }}_{3}e^{\lambda_{5}x}+\lambda_{6}\alpha_{6}{\vec{\nu }}_{3}e^{\lambda_{6}x}$$

The boundary conditions can be either explicit (open traxels with a prescribed voltage or current) or implicit (meandering traxels with a connected floating voltage and current); these types are illustrated in Figure 5.

#### 2.2.1. Explicit Boundary Conditions

For the explicit boundary conditions, a traxel either has a prescribed voltage for a traxel, which is an in- or output, or a prescribed current of 0 for a traxel that is not connected:

(26) $${\hat{U}}_{n}\left(\xi ,\omega \right)={\hat{U}}_{\mathrm{in}/\mathrm{out}}$$

(27) $${\left.\frac{\partial {\hat{U}}_{n}\left(x,\omega \right)}{\partial x}\right|}_{x=\xi }=0$$

where ξ=0∨L.

Each traxel is given two equations to solve for the coefficients, one at the input x=0 and one at the output x=L. Accordingly, for *N* traxels, there are 2N equations and, hence, also 2N eigenvalues $\lambda_{i}$, eigenvectors ${\vec{\nu }}_{i}$, and coefficients $\alpha_{i}$. Recalling the expression for the voltage (Equation (18)), we find, for the explicit voltage boundaries:

(28) $${\hat{U}}_{i}\left(0,\omega \right)=\alpha_{1}{\vec{\nu }}_{1,i}+\sum_{k=3}^{6}\alpha_{k}{\vec{\nu }}_{k,i}$$

(29) $${\hat{U}}_{i}\left(L,\omega \right)=\alpha_{1}{\vec{\nu }}_{1,i}+\alpha_{2}{\vec{\nu }}_{2,i}L+\sum_{k=3}^{6}\alpha_{k}{\vec{\nu }}_{k,i}e^{\lambda_{k}L}$$

and for the current-type boundaries:

(30) $${\hat{I}}_{i}\left(0,\omega \right)=0=\alpha_{2}{\vec{\nu }}_{2,i}+\sum_{k=3}^{6}\alpha_{k}{\vec{\nu }}_{k,i}\lambda_{k}$$

(31) $${\hat{I}}_{i}\left(L,\omega \right)=0=\alpha_{2}{\vec{\nu }}_{2,i}+\sum_{k=3}^{6}\alpha_{k}{\vec{\nu }}_{k,i}\lambda_{k}e^{\lambda_{k}L}$$

#### 2.2.2. Implicit Boundary Conditions

At the point where a traxel meanders, like in Figure 9, neither the current (non-zero) nor voltage (the voltage is not determined by an external source) are known. However, the meandering does provide implicit boundary conditions (where ξ=0∨L):

(32) $$\begin{array}{cc} {\hat{U}}_{i}\left(\xi ,\omega \right) & ={\hat{U}}_{i+1}\left(\xi ,\omega \right)\equiv {\hat{U}}_{i,i+1} \end{array}$$

(33) $$\begin{array}{cc} {\hat{I}}_{i}\left(\xi ,\omega \right) & =-{\hat{I}}_{i+1}\left(\xi ,\omega \right)\Rightarrow {\left.\frac{\partial {\hat{U}}_{i}\left(x,\omega \right)}{\partial x}\right|}_{x=\xi }=-{\left.\frac{\partial {\hat{U}}_{i+1}\left(x,\omega \right)}{\partial x}\right|}_{x=\xi } \end{array}$$

We have two unknowns (*U* and *I*) per meander, but we also have two equations for each pair of meandered traxels. Thus, for $M_{1}$, the voltage-type implicit boundaries become:

(34) $$\begin{array}{cc} {\hat{U}}_{i}\left(L,\omega \right) & ={\hat{U}}_{i+1}\left(L,\omega \right) \end{array}$$

(35) $$\begin{array}{cc} \alpha_{1}{\vec{\nu }}_{1,i}+\alpha_{2}{\vec{\nu }}_{2,i}L+\sum_{k=3}^{6}\alpha_{k}{\vec{\nu }}_{k,i}e^{\lambda_{k}L} & =\alpha_{1}{\vec{\nu }}_{1,i+1}+\alpha_{2}{\vec{\nu }}_{2,i+1}L+\sum_{k=3}^{6}\alpha_{k}{\vec{\nu }}_{k,i+1}e^{\lambda_{k}L} \end{array}$$

Rewriting the above yields the implicit voltage boundary condition:

(36) $$\alpha_{1}\left({\vec{\nu }}_{1,i}-{\vec{\nu }}_{1,i+1}\right)+\alpha_{2}\left({\vec{\nu }}_{2,i}-{\vec{\nu }}_{2,i+1}\right)L+\sum_{k=3}^{6}\alpha_{k}\left({\vec{\nu }}_{k,i}-{\vec{\nu }}_{k,i+1}\right)e^{\lambda_{k}L}=0$$

The currents at both boundaries are equal in size and opposite of sign:

(37) $$\begin{array}{cc} {\hat{I}}_{i}\left(L,\omega \right) & =-{\hat{I}}_{i+1}\left(L,\omega \right) \end{array}$$

(38) $$\begin{array}{cc} \alpha_{2}{\vec{\nu }}_{2,i}+\sum_{k=3}^{6}\alpha_{k}{\vec{\nu }}_{k,i}\lambda_{k}e^{\lambda_{k}L} & =-\alpha_{2}{\vec{\nu }}_{2,i+1}-\sum_{k=3}^{6}\alpha_{k}{\vec{\nu }}_{k,i+1}\lambda_{k}e^{\lambda_{k}L} \end{array}$$

Rewriting the above yields the implicit current boundary condition:

(39) $$\alpha_{2}\left({\vec{\nu }}_{2,i}+{\vec{\nu }}_{2,i+1}\right)+\sum_{k=3}^{6}\alpha_{k}\left({\vec{\nu }}_{k,i}+{\vec{\nu }}_{k,i+1}\right)\lambda_{k}e^{\lambda_{k}L}=0$$

The same steps can be followed for meanders $M_{2}$ in the left hand side in Figure 9 (at x=0), for which the implicit voltage boundary condition becomes:

(40) $$\alpha_{1}\left({\vec{\nu }}_{1,i}-{\vec{\nu }}_{1,i+1}\right)+\sum_{k=3}^{6}\alpha_{k}\left({\vec{\nu }}_{k,i}-{\vec{\nu }}_{k,i+1}\right)=0$$

Additionally, for which the implicit current boundary condition becomes:

(41) $$\alpha_{2}\left({\vec{\nu }}_{2,i}+{\vec{\nu }}_{2,i+1}\right)+\sum_{k=3}^{6}\alpha_{k}\lambda_{k}\left({\vec{\nu }}_{k,i}+{\vec{\nu }}_{k,i+1}\right)=0$$

For a meandering traxel, one can use either the current condition or voltage condition. A systematic method is required, because both have to be used and each traxel needs one condition on each side, e.g., upper traxel uses the voltage condition and the lower traxel uses the current condition. Table 1 presents an overview of the different boundary conditions.

#### 2.2.3. Applying Boundary Conditions

The boundary conditions from a three traxel sample with open ends are used to solve for $\alpha_{i}$. Equation (25) can be written into a matrix form. For the geometry, track 1 is the upper, 2 the middle, and 3 the lower track. On the left of track 1, a voltage $U_{\mathrm{in}}=1V$ is applied, and, on the right of track 3, a ground is connected $U_{\mathrm{out}}=0V$, the currents have undefined values on these two boundaries. For all of the other boundaries, the current is taken to be 0 A (open boundaries) and the voltage is taken as an undefined variable.

The matrix form then becomes, with the upper row, the equation for ${\hat{U}}_{1}\left(0,\omega \right)$ and the lower row for ${\hat{U}}_{3}\left(L,\omega \right)$:

(42) $$\left[\begin{array}{cccccc} 1 & 0 & -1 & -1 & 1 & 1\\ 0 & 1 & -\lambda_{3}e^{\left(\lambda_{3}L\right)} & -\lambda_{4}e^{\left(\lambda_{4}L\right)} & \lambda_{5}e^{\left(\lambda_{5}L\right)} & \lambda_{6}e^{\left(\lambda_{6}L\right)}\\ 0 & 1 & 0 & 0 & -2\lambda_{5} & -2\lambda_{6}\\ 0 & 1 & 0 & 0 & -2\lambda_{5}e^{\left(\lambda_{5}L\right)} & -2\lambda_{6}e^{\left(\lambda_{6}L\right)}\\ 0 & 1 & \lambda_{3} & \lambda_{4} & \lambda_{5} & \lambda_{6}\\ 1 & L & e^{\left(\lambda_{3}L\right)} & e^{\left(\lambda_{4}L\right)} & e^{\left(\lambda_{5}L\right)} & e^{\left(\lambda_{6}L\right)} \end{array}\right]\left[\begin{array}{c} \alpha_{1}\\ \alpha_{2}\\ \alpha_{3}\\ \alpha_{4}\\ \alpha_{5}\\ \alpha_{6} \end{array}\right]=\left[\begin{array}{c} U_{\mathrm{in}}\\ 0\\ 0\\ 0\\ 0\\ U_{\mathrm{out}} \end{array}\right]$$

Taking the inverse of the matrix and multiplying it with the vector with boundary conditions yields the coefficients. The horizontal current component can then be found from Equation (8). The total impedance of the system can be found by dividing the voltage difference between both ends by the input current:

(43) $${\hat{Z}}_{\mathrm{total}}\left(\omega \right)=\frac{{\hat{U}}_{1}\left(0,\omega \right)-{\hat{U}}_{3}\left(L,\omega \right)}{{\hat{I}}_{1}\left(0,\omega \right)}$$

In the case meandering boundaries are studied, Equations (2)–(5) in the matrix above are replaced by Equations (36) and (39)–(41), respectively (sticking to the convention).

Finally, the average dissipated power is important for the experimental validation presented in [42], and it can be expressed as (with * the complex conjugated):

(44) $$P=\frac{1}{2}ℜ\left\{UI^{*}\right\}=\frac{1}{2}ℜ\left\{\frac{U\;\cdot \;U^{*}}{Z_{\mathrm{total}}}\right\}$$

Extensions of the total model can be found in Appendix B.

### 2.3. Dimensionless Parameters and Limit Cases

For the moment, we turn to dc voltages to obtain some insight in the complex conduction properties of multi-traxel prints. Current can flow via the shortest path: bulk conduction, the case where the contact properties dominate the conduction; can flow following the traxels: traxel conduction, the case where the bulk properties dominate the conduction; or, somewhere in between: mixed conduction, as will be discussed in the Section 4. The boundary conditions in combination with three determining factors or dimensionless parameters can express which situation occurs: the anisotropy ratio, the aspect ratio, and the number of traxels. The anisotropy ratio expresses the material effects, the aspect ratio, the geometry effects, and the number of traxels related to the discrete print design. The upcoming sections explain the details and use of the parameters.

To arrive to the choice of these parameters, one can study the ratio between the vertical resistance and horizontal resistance of a sample in a dc case. This resistance ratio presents the combined effect of the geometry, material properties, and printing design:

(45) $$RR=\frac{R_{\mathrm{vert}.}}{R_{\mathrm{hor}.}}=\frac{\frac{(N-1)W\rho }{HL}+\frac{(N-1)\sigma }{HL}}{\frac{\rho L}{NHW}}=\underset{\mathrm{Discretization}}{\underset{︸}{\frac{N-1}{N}}}\underset{\mathrm{Geometry}}{\underset{︸}{{\left(\frac{NW}{L}\right)}^{2}}}\underset{\mathrm{Material}}{\underset{︸}{\frac{\rho +\frac{\sigma }{W}}{\rho }}}$$

This ratio relates the shape of the voltage distribution to an isotropic voltage distribution without contacts in a rectangular sample (while assuming dc conditions). A value of 1 presents an equivalent isotropic voltage distribution, whereas values bigger than 1 suggest a voltage drop from top to bottom and smaller than 1 a voltage drop from left to right. In other words, it provides a measure for how the voltage will distribute over the traxels, depending on the electrical properties and geometry.

#### 2.3.1. Anisotropy Ratio

The anisotropy ratio is defined as Γ in Equation (13), which provides greater insight into the influence of the material or electrical parameters and how they influence the type of conduction:

(46) $$\Gamma =\frac{\left(\frac{\rho }{1+j\omega \rho \epsilon_{0}\epsilon_{r}}\right)}{\left(\frac{\rho }{1+j\omega \rho \epsilon_{0}\epsilon_{r}}\right)+\left(\frac{\sigma /W}{\left(1+j\omega \sigma C_{0}\right)}\right)}$$

From this expression, it becomes clear that Γ≤1, because all the physical parameters ≥0. Γ is defined as the anisotropy ratio, since it gives the ratio of impedivity (complex resistivity) in both directions. Therefore, it provides a measure as to how the horizontal bulk properties relate to the combination of vertical bulk and surface properties. This is similar to the definition of the anisotropy ratio by [35]. In this work, they define the anisotropy ratio as the resistivity in vertical direction divided by the resistivity in the horizontal direction, with homogenized resistivity terms.

Several limit cases can be defined for Γ. For low frequencies, the purely resistive nature of Γ dominates and Γ can be approximated, as follows:

(47) $$\Gamma_{\mathrm{DC}}=\lim_{\omega \to 0}\Gamma \left(\omega \right)=\frac{\left(\frac{\rho }{1}\right)}{\left(\frac{\rho }{1}\right)+\left(\frac{\sigma /W}{1}\right)}=\frac{\rho }{\rho +\sigma /W}$$

This term can be recognized in Equation (45) as the material term. For the resistive case, the value of $\Gamma_{\mathrm{DC}}$ determines the conductive behavior. If σ≫ρW, i.e., when $\Gamma_{\mathrm{DC}}$ becomes small, there will only be traxel conduction. For σ≪ρW, i.e., when $\Gamma_{\mathrm{DC}}\approx 1$, the current will take the shortest path crossing the traxels. In the case of dc conduction, isotropic conduction takes place when ρ is isotropic and there is no contact resistance. In that case, there are no material effects:

(48) $$\Gamma_{\mathrm{isotropic}}=1$$

A high frequency limit also exists; for high frequencies, the capacitive effects become dominant instead of the resistive terms:

(49) $$\lim_{\omega \to \infty }\Gamma \left(\omega \right)=\frac{\left(\frac{\rho }{j\omega \rho \epsilon_{0}\epsilon_{r}}\right)}{\left(\frac{\rho }{j\omega \rho \epsilon_{0}\epsilon_{r}}\right)+\left(\frac{\sigma /W}{\left(j\omega \sigma C_{0}\right)}\right)}=\frac{\left(\frac{1}{\epsilon_{0}\epsilon_{r}}\right)}{\left(\frac{1}{\epsilon_{0}\epsilon_{r}}\right)+\left(\frac{1}{C_{0}W}\right)}=\frac{C_{0}W}{C_{0}W+\epsilon_{0}\epsilon_{r}}$$

Hence, at high frequency, Γ gives the ratio of contact capacitance over the combination of bulk capacitance and contact capacitance. In case Γ≈1, the contact capacitance is dominant and bulk conduction occurs (again isotropic conduction occurs). In case Γ≪1, the bulk capacitance is dominant and traxel conduction occurs (since a higher capacitance yields lower total impedance).

#### 2.3.2. Number of Traxels

The discretization term presented in Equation (45) also shows the importance of the number of traxels *N*, where the number of traxels influences the electrical conduction. Therefore, *N* is also used as the determining factor. The contact resistance becomes more dominant in the case of thin traxels or large *N*. The second half of the general 2D-model presented in Equation (17) can be expressed as a partial derivative when *W* becomes close to 0:

(50) $$\lim_{W\to 0}\frac{\left({\hat{U}}_{n-1}\left(x,\omega \right)-2{\hat{U}}_{n}\left(x,\omega \right)+{\hat{U}}_{n+1}\left(x,\omega \right)\right)}{W^{2}}=\frac{\partial^{2}\hat{U}\left(x,y,\omega \right)}{\partial y^{2}}$$

In this case, the PDE would resolve into the Laplacian for homogeneous anisotropic materials:

(51) $$\frac{\partial^{2}\hat{U}\left(x,y,\omega \right)}{\partial x^{2}}+\Gamma \left(\omega \right)\frac{\partial^{2}\hat{U}\left(x,y,\omega \right)}{\partial y^{2}}=0$$

This indicates the generality of the model, since, for homogeneous material properties, it has the Laplace equation for orthotropic materials as a result, being defined as [43]:

(52) $$\frac{1}{\rho_{x}}\frac{\partial^{2}\phi }{\partial x^{2}}+\frac{1}{\rho_{y}}\frac{\partial^{2}\phi }{\partial y^{2}}=0$$

This shows the meaning of Γ in the limit of homogeneous, anisotropic media, where it gives the ratio of the orthogonal resistivities.

#### 2.3.3. Aspect Ratio

For high numbers of traxels, the aspect ratio in Equation (53), provided the ratio between the sample width and sample length, can be used to express the effects of the geometry on the conduction, and it can be recognized inside the squared geometry term in Equation (45):

(53) $$AR=\frac{NW}{L}$$

Only for a large number of layers (where the number of contacts is approximately equal to the number of traxels: $\frac{N-1}{N}\approx 1$), the number of traxels does not influence the electrical conduction. The resistance ratio then becomes:

(54) $$RR{|}_{N\gg 1}={\left(\frac{NW}{L}\right)}^{2}\frac{\rho +\sigma /W}{\rho }=AR^{2}\frac{1}{\Gamma_{\mathrm{DC}}}$$

Hence, the effect of the aspect ratio and anisotropy ratio on the conduction/voltage distribution can counter each other. For slender structures, it is expected that a very low anisotropy ratio will significantly change the mode of conduction.

#### 2.3.4. Open-Ended Resistance Approximation

Because the resistance ratio combines the effect of the aspect ratio, anisotropy ratio, and the number of traxels, these parameters are difficult to separately analyze. Therefore, the other three dimensionless numbers will be used for further model analysis. It is also useful to have a simplified expression for the total resistance that is based on these three dimensionless numbers for further analysis.

The total resistance can be approximated by assuming the different components as lumped resistances (similar to what was done with expressing the resistance ratio). This only holds for limit cases, where a certain resistance component is dominant: very large aspect ratios, very small aspect ratio, or very large contact resistance. For the open-ended case, where traxels are not connected to each other, one then considers the horizontal, the vertical, and the inter-traxel resistance components:

(55) $$R_{\mathrm{open}}\approx R_{\mathrm{hor}.}+R_{\mathrm{vert}.}+R_{\mathrm{inter}-\mathrm{traxel}}=\frac{\rho L}{NWH}+\frac{\rho (N-1)W}{HL}+\frac{(N-1)\sigma }{HL}$$

These can be rewritten in terms of the aspect ratio AR and anisotropy ratio $\Gamma_{\mathrm{DC}}$:

(56) $$R_{\mathrm{open}}\approx \frac{\rho }{H}\left(\frac{1}{AR}+\frac{N-1}{N}\frac{AR}{\Gamma_{\mathrm{DC}}}\right)$$

This function contains both a direct and an inverse proportionality with the aspect ratio. This can be recognized in both the actual model results and FEM data shown in Figure 14. This becomes more evident in the case of large numbers of traxels N≫1 and anisotropy ratios that are close to 1 (so also low σ); in that case, Equation (56) reduces to:

(57) $$R_{\mathrm{open}}{|}_{N\gg 1,\Gamma_{\mathrm{DC}}\approx 1}\approx \frac{\rho }{H}\left(\frac{1}{AR}+AR\right)$$

This shows the equivalence between large and small aspect ratios for the resistance. The resistance is the same for an aspect ratio of 1/100 as it is for 100, namely $\frac{\rho }{H}\left(1/100+100\right)$. This can be recognized in the symmetry of the resistance around an aspect ratio of 1 for cases with low inter-traxel resistance (e.g., Figure 14).

#### 2.3.5. Meandering Resistance Approximation

The effect of the boundary conditions needs to be captured in order to approximate the resistance of the meandering case. The meandering case can be considered as two parallel resistances: the open-ended/bulk conduction limit case in parallel to the traxel conduction limit case. The traxel conduction limit case is simply the resistance when following the meandering path of all traxels ($R_{\mathrm{traxel}}=\frac{N\rho L}{WH}=\frac{\rho }{H}\frac{N^{2}}{AR}$):

(58) $$R_{\mathrm{meander}}\approx \frac{1}{\frac{1}{R_{\mathrm{open}}}+\frac{1}{R_{\mathrm{traxel}}}}=\frac{1}{\frac{1}{\frac{\rho }{H}\left(\frac{1}{AR}+\frac{N-1}{N}\frac{AR}{\Gamma_{\mathrm{DC}}}\right)}+\frac{1}{\frac{N\rho L}{WH}}}=\frac{\rho }{H}\frac{N^{2}\left(AR^{2}\left(N-1\right)+N\Gamma_{\mathrm{DC}}\right)}{AR(AR^{2}\left(N-1\right)+N\left(N^{2}+1\right)\Gamma_{\mathrm{DC}})}$$

Now, in the case where the anisotropy ratio is close to 1 and the number of traxels *N* approaches infinity, we then find the expression for the open-ended resistance with an anisotropy ratio close to 1:

(59) $$\lim_{N\to \infty }R_{\mathrm{meander}}{|}_{N\gg 1,\Gamma_{\mathrm{DC}}\approx 1}=\frac{\rho }{H}\frac{1+{AR}^{2}}{AR}=\frac{\rho }{H}\left(\frac{1}{AR}+AR\right)=R_{\mathrm{open}}{|}_{N\gg 1,\Gamma_{\mathrm{DC}}\approx 1}$$

Hence, this shows that, for large numbers of traxels and insignificant inter-traxel resistance, the resistance becomes the same for the open-ended and meandering cases. Accordingly, the anisotropy ratio, aspect ratio, and number of traxels can be used to approximate the total resistance for limit cases. It has to be noted that, with these definitions, the dimensionless numbers are not completely independent, since they all contain either *W* or *N* in their definition.

## 3. Methods

### 3.1. Model Implementation

**Figure 10:**
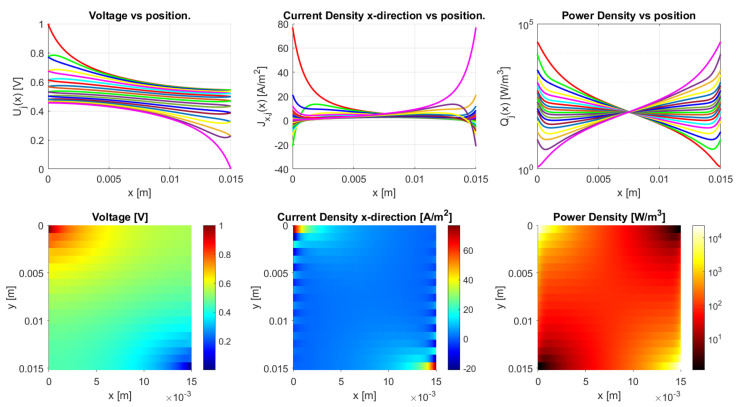
The results of the Matlab implementation of the model, showing the voltage, current density in *x*-direction, and the power density. The values presented in Table 2 are used as the model parameters, and traxels are modeled to be meandering at x=0 (except for traxel 1, **top**) and x=L (except for traxel 19, **bottom**). This yields an anisotropy ratio of Γ=0.528, indicating a small amount of anisotropic conduction.

The model is implemented in Matlab (Matlab R2020a) for the general dc case. It works for an arbitrary number of *N*-traxels, having Equation (18) run from i=1 to 2N, where the bulk and contact properties are both implemented. The code has the possibility of defining the boundary conditions by means of a vector for the left boundaries and a vector for the right boundaries. Boundaries can be open, connected to other traxels, have input voltages, and be grounded. The code then calculates the voltages, current density, and power dissipation for every traxel as well as the total resistance. The power dissipation is calculated by combining the power dissipation inside the traxels with the power dissipation through the vertical bulk and contacts. For large aspect ratios, there is a risk of the numerical model becoming singular, primarily due to quick growth (positive λ) or a decrease (negative λ) of the exponential terms. The traxel calculations are implemented with x=0 positioned at the center of the traxels to reduce the chances of this happening. Accordingly, the exponents are rewritten as $e^{\pm \lambda_{i}L/2}$ for calculating the coefficients with the boundary conditions. The model code is open-access and it can be found in [46] as indicated in the Supplementary Materials section. Figure 10 represents the code output, with the voltage, current density in *x*-direction, and power dissipation density being given in graphs for each traxel (top row) and color plots of the distributions (bottom row).

The model can be verified by comparing it to the limit cases from the lumped resistance approximation. This only works for situations with large and small aspect ratios and low anisotropy ratios, since the resistance approximation expresses the bulk conduction as horizontal and vertical in series (neglecting the PDE behavior of conduction). Therefore, FEM simulations are required for the full verification.

### 3.2. FEM Simulations

**Figure 11:**
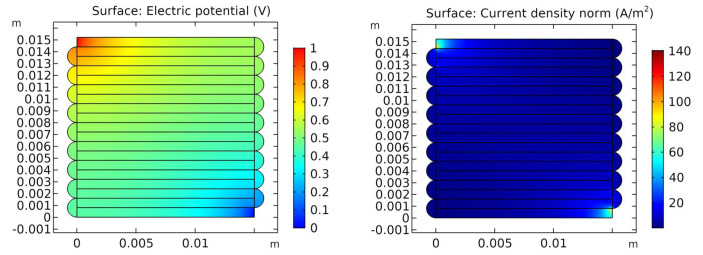
The FEM simulation results in COMSOL with the parameters from Table 2. The left figure presents the voltage distribution and the right one shows the current density norm.

The structures are simulated by the Finite Element Method (FEM) using the Electric Currents module of COMSOL, following the methodology presented in [42]. The voltages, currents, and power dissipation density, as well as the total impedance, are simulated. The electrical properties are implemented in COMSOL through the material properties and contact impedance functionality. The FEM model has already been validated with a mesh convergence study and experiments for the dc-case in a previous study [42]. The parameters from the simulations in this study are used for the verification and analysis of the model, as in Table 2. Figure 11 presented an example of the simulation results.

The model verification is performed by comparing the overall resistance values, as well as by comparing the traxel voltage and current densities between the simulation and model results. During the verification, several shortcomings of the model and FEM simulations have to be taken into account. The FEM simulations generate full 2D-solutions, as compared to the lumped traxel solutions of the model. Furthermore, the FEM simulations include the meander geometry, instead of connecting the voltage and currents at the ends of the traxels. On the other hand, the model resolution is not limited due to the analytical expression for each traxel, whereas the FEM simulations need discretization. In order to verify the model for these type of shortcomings, the total resistance of the model and simulations are compared as a function of the three dimensionless parameters put forth in the previous section: the anisotropy ratio Γ, the aspect ratio AR, and the number of traxels *N*. In this way, the model can be verified for a large range of parameters, both with open-ended traxels and meandering traxels.

### 3.3. Model Analysis

The model analysis is performed on the total resistance with the three dimensionless numbers, where the dc anisotropy ratio is used from now on, and it is simply referred to as Γ. The effects of these dimensionless numbers, as well as open-ended and meandering traxels, are studied. Some limit case parameter sets are used to show the difference between isotropic and anisotropic conduction. Finally, application constriction-resistive strain sensors are modeled. All of the above analysis steps are performed by means parameter sweeps in the Matlab implementation of the model.

## 4. Results

The model is first verified through simplified analytical models and FEM simulations. Readers who are interested in the model results are referred to Section 4.2.

### 4.1. Model Verification

For verification, the model results are compared to the FEM simulations in COMSOL in three different steps:

- A comparison of voltage and *x*-current density for both an open-ended and meandering sample with three traxels. This example is used to clearly show the basics and compare the results. The $\sigma =2\;\times \;10^{-2}\;\Omega m^{2}$ used yields an anisotropy ratio of 0.1007, which is well suited for anisotropic conduction.
- A comparison of the total resistance for a sample with the parameters from Table 2 with, in one case, a varying aspect ratio and, in the other case, a varying anisotropy ratio. This can be used to study the model in the range for which the FEM simulations are experimentally validated, and to give an indication of the shortcomings of the model. Furthermore, a comparison is made to the approximated resistance expressions for both the open-ended and meandering case as an analytical verification of the limit cases.
- A comparison of the total resistance for the combined aspect ratio and anisotropy ratio for different numbers of traxels, in order to verify the model over the entire range of interest. A low, medium, and large number of traxels are used of, respectively, 5, 19, and 51 traxels.

#### 4.1.1. Three Traxel Verification

**Figure 12:**
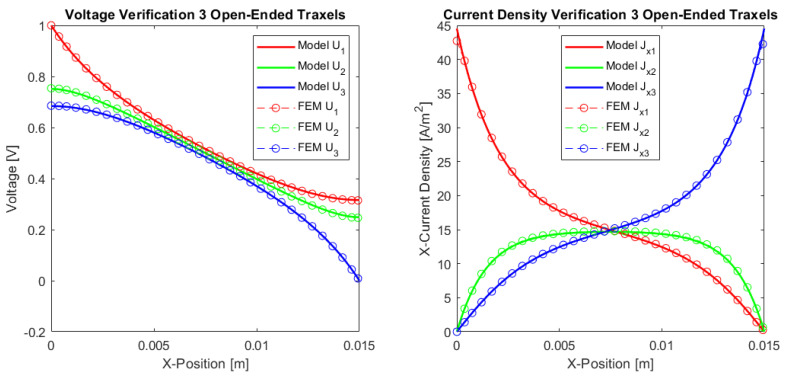
Comparison between the model and simulations of the dc voltage and current density in *x*-direction in the traxels of a sample with three traxels with open ends. The parameters shown in Table 2 are used and the number of traxels, N=3, and inter-traxel resistance, $\sigma =2\;\times \;10^{-2}\;\Omega m^{2}$, are changed. The total resistance from the model is 140.3 kΩ, which gives an error of 0.53% with the FEM simulation.

**Figure 13:**
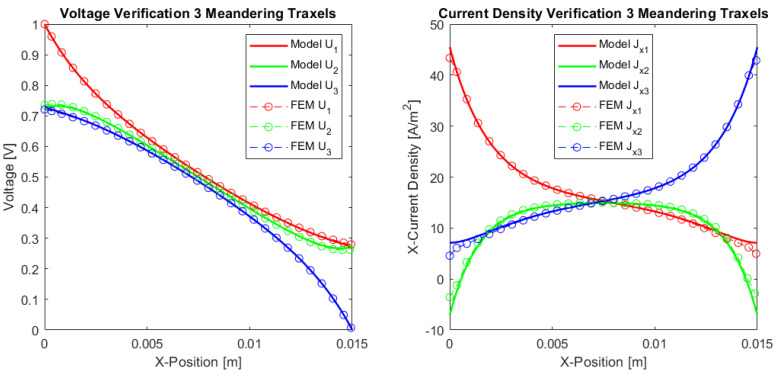
A comparison between the model and simulations of the dc voltage and current density in *x*-direction in the traxels of a sample with three traxels with meandering ends. The parameters listed in Table 2 are used and the number of traxels, N=3, and inter-traxel resistance, $\sigma =2\;\times \;10^{-2}\;\Omega m^{2}$, are changed. The total resistance from the model is 137.4 kΩ, which gives an error of 0.23% with the FEM simulation.

Figure 12 presents the verification results of the three traxels for the open-ended case and in Figure 13 for the meandering traxels. In both cases, the model and FEM results only show small differences, with errors in the total resistance as compared to the FEM simulations of 0.53% for the open-ended case and 0.23% for the meandering case. The anisotropy ratio used is Γ=0.1007 and the aspect ratio used is AR=0.16. Therefore, the voltage drop is mainly from left to right and not from top to bottom due to the anisotropy and long structure. In the meandering case, the voltages at the ends of the traxels are clearly the same as for the connected ends in the model case. In the FEM simulation, there is a small gap present between the voltages at the meandering ends, since it takes the added resistance of the meandering part and the 2D conduction into account. This can also be recognized in the results for the current density at the ends. Overall, the verification for the three traxels shows that, for these specific parameters, the results between the model and FEM fit well, and that the main errors are posed by the 2D-conduction and lack of resistance of the meandering parts.

#### 4.1.2. Anisotropy Ratio and Aspect Ratio Verification

**Figure 14:**
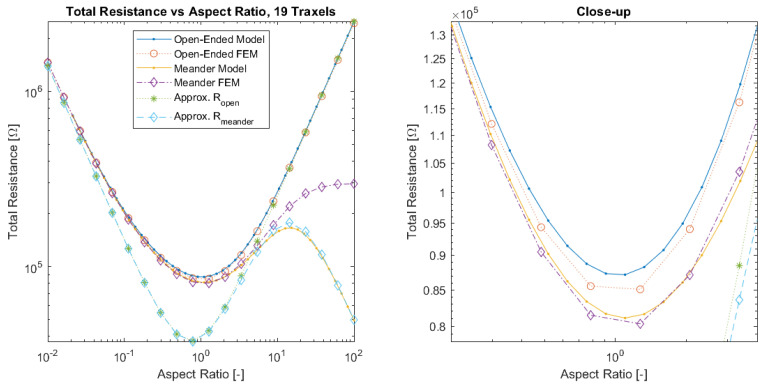
A comparison of the total resistance as function of aspect ratio for open-ended and meandering traxels between the model, FEM simulation, and resistance approximation results. Errors between model and FEM results occur at an aspect ratio around 1 due to 2D-conduction effects and for the meandering case for large aspect ratios due to the increased importance of the resistance of the meandering parts.

For a next generalization step, the parameters that are shown in Table 2 are used to verify the model by individually sweeping over the traxel length *L* and inter-traxel resistance σ to study both the effect of the aspect ratio AR and anisotropy ratio Γ and comparing the total resistance. A comparison is made between the model results, FEM simulations results, and the approximated resistance expressions from Section 2.3.4. Figure 14 presents the total resistance as function of aspect ratio for the model, FEM simulations, and resistance approximation with open-ends and meandering traxels. The main errors again occur for aspect ratios around 1, since 2D-conduction inside traxels is more important, and for high aspect ratios in the meandering case, since the resistance of the meandering ends cannot be neglected anymore. The total resistance as function of aspect ratio has a parabolic-like shape, since for both big and small aspect ratios the resistance goes up for the respective vertical and horizontal resistance component in the case of an anisotropy ratio close to 1, as can also be seen in Equation (56). For the meandering case, the resistance drops again for high aspect ratios, since the traxel conduction becomes more significant.

**Figure 15:**
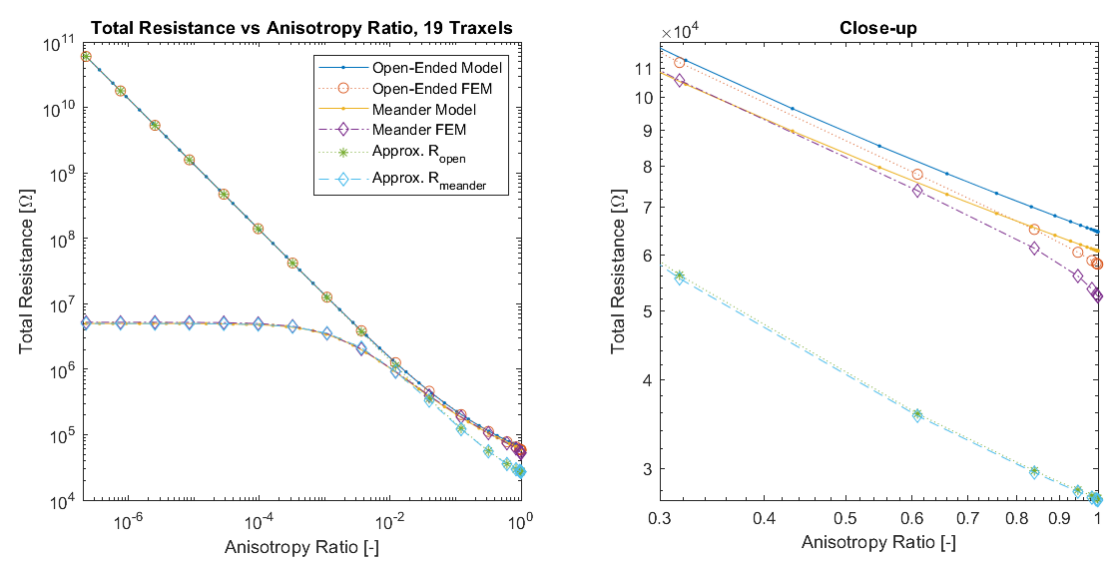
A comparison of the total resistance as a function of anisotropy ratio for open-ended and meandering traxels between the model, FEM simulation, and resistance approximation results. The errors between the model and FEM results occur at an anisotropy ratio that is close to 1 due to 2D-conduction effects.

Figure 15 presents the total resistance as function of anisotropy ratio for the model, FEM simulations, and resistance approximation with open-ended and meandering traxels. The main errors in both the model and resistance approximation occur for anisotropy ratios that are close to 1, due to the lumping of the conduction in the *y*-direction, since the 2D-effects of bulk conduction within traxels become important in that range. The sweep over the anisotropy ratio also reveals the large difference in resistance for low anisotropy ratios between the open-ended and meandering case. For low anisotropy ratios, the total resistance of the meandering case becomes constant, since purely traxel conduction occurs and a further change in anisotropy ratio does not have an effect on traxel conduction.

#### 4.1.3. Total Resistance Verification

**Figure 16:**
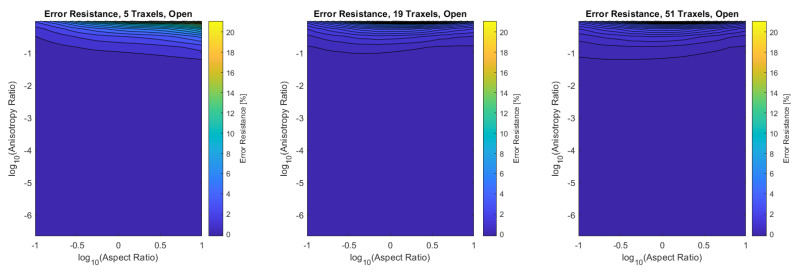
The error of the total resistance for the open-ended case with different numbers of traxels. The RMS-error for the different numbers of traxels is, respectively, 4.75%, 2.51%, and 1.97%.

**Figure 17:**
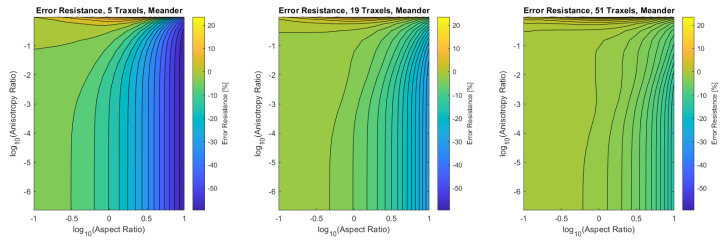
The error of the total resistance for the meandering case with different numbers of traxels. The RMS-error for the different numbers of traxels is, respectively, 24.33%, 10.26%, and 4.70%.

Finally, the verification is performed for the combined dimensionless parameters, for a low (5), medium (19), and high number of traxels (51) by sweeping over the traxel length and the inter-traxel resistance. The error in the modeled total resistance as compared to the FEM simulations is presented in Figure 16 for the open-ended case and in Figure 17 for the meandering case.

**Figure 18:**
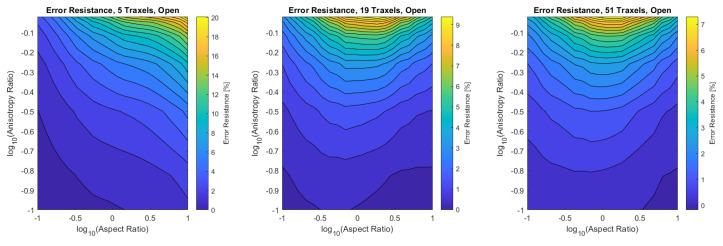
A close-up of the data in Figure 16 for anisotropy ratios close to 1.

In the open-ended case, the error is only significant for anisotropy ratios that are close to 1, as can be seen more clearly in the close-up shown in Figure 18. This is due to 2D-conduction effects, since bulk conduction is dominant with 2D-conduction inside the traxels. There is only little influence on the error from the aspect ratio for the entire range of dimensionless numbers.

**Figure 19:**
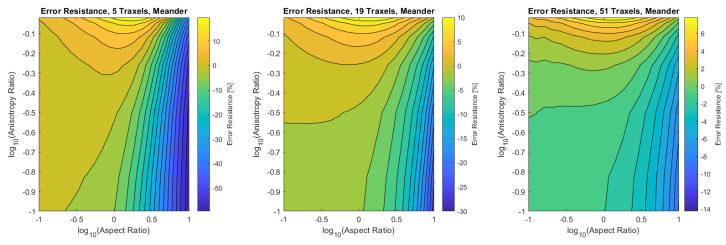
A close-up of the data in Figure 17 for anisotropy ratios close to 1.

In the meandering case, on the other hand, the error is more significant for both anisotropy ratios close to 1 and for big aspect ratios as can be seen more clearly in the close-up in Figure 19. Again, this is due to 2D-effects, but also because of neglecting the resistance of the meandering parts in the model.

For both the meandering and open-ended case, the effect of anisotropy ratios below Γ<0.01 on the error is insignificant. The root-mean-square (RMS) error has been calculated for all cases and can be found in Table 3. It becomes clear that larger numbers of traxels give a lower error in the model, and, furthermore, the open-ended case can be modeled with smaller errors. The error plots with RMS errors have also been calculated for the resistance approximation, and these can be found in Appendix C.

All-in-all, a verification has been performed on a traxel level and on the total resistance. The total resistance has been verified over a big range of anisotropy ratio, aspect ratio, and number of traxels. It was shown that the 2D-conduction effects and neglecting the meander resistance are the main causes for errors, as was expected. For the open-ended case, the model is reliable in all cases where the anisotropy ratio is approximately below Γ<0.5, giving an error of approximately 10% for five traxels. For the meandering case, the error strongly depends on the aspect ratio and the number of traxels. For aspect ratios that are close to 1, low numbers of traxels, and for big aspect ratios, the error becomes large due to 2D-conduction effects and neglecting meander resistance.

### 4.2. Model Findings

This subsection presents the findings of the model. First, the general findings are presented, after which the effects from the anisotropy ratio, the aspect ratio, the number of traxels, the presence of meanders, and the presence of multiple inputs are given.

#### 4.2.1. General Model Findings

Figure 10 shows the results of the model with the parameters from Table 2, presenting the voltage, current density in *x*-direction, and power density data. From these parameters an anisotropy ratio of Γ=0.528 is found. This indicates a small amount of anisotropic conduction that can also be seen in the voltage distribution, which was already discussed in [42]. This particular example has meandering ends, which yields some differences relative to the open-ended case.

The differences between open-ended and meandering ended case can be recognized in Figure 12 and Figure 13. For the open-ended case, the voltages at the ends are not equal for neighbouring traxels, whereas they are for the meandering case. Furthermore, for the meandering case, the current density in the x-direction can change direction at the meandering ends, whereas, for the open-ended case, the current density in the x-direction is always 0 A/m${}^{-2}$ at the floating ends. Figure 10 presents the inversion of the current direction.

In Figure 10, the top right graph presents the power density in every traxel. It becomes clear that, for most traxels, the power density is the highest at the edges for the meandering case. The meandering ends cause hot spots at the edges of the samples, due to the combination of current inversion between traxels at the edges and due to the lack of contact resistance in the meanders themselves, since the meanders link the traxels without an inter-traxel resistance. These hot spots were already recognized in FEM simulations and the experiments in [42]. They become more pronounced for low anisotropy ratios and high aspect ratios, since, in these cases, traxel conduction occurs. This graph, combined with the research from [42], also indicates that the power density in, for example, Figure 10 can be used for the validation of model cases through measuring the thermal distribution experimentally. As was already shown in [42], for low inter-traxel resistance, the power dissipation and heating is very inhomogeneous and mainly close to the contacts, whereas the heating is very homogeneous for high inter-traxel resistance.

#### 4.2.2. Anisotropy Ratio

Three modes of conduction exist, depending on the anisotropy ratio: bulk, mixed, and traxel conduction, as discussed in Section 2.3.1. The model results are calculated for varying anisotropy ratios with three different inter-traxel resistance values of $\sigma =2\;\times \;10^{-4}\;\Omega m^{2}$, $\sigma =2\;\times \;10^{-2}\;\Omega m^{2}$, and $\sigma =2\;\times \;10^{-1}\;\Omega m^{2}$ to represent these three types of conduction. For these calculations, square geometries are used to exclude the effect of the aspect ratio on the conduction.

**Figure 20:**
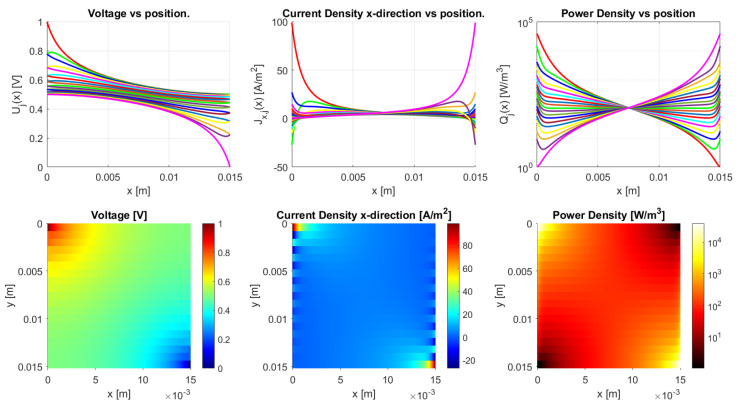
The model results for a meandering sample with the parameters from Table 2 and an inter-traxel resistance of $\sigma =2\;\times \;10^{-4}\;\Omega m^{2}$. This gives an anisotropy ratio of 0.918, showing bulk conduction.

**Figure 21:**
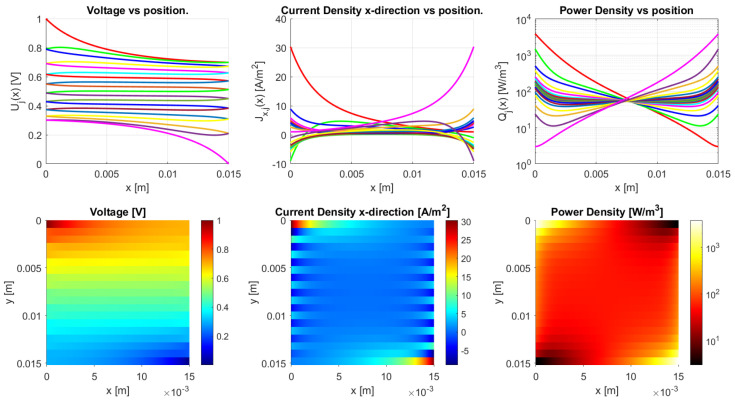
Model results for a meandering sample with the parameters from Table 2 and an inter-traxel resistance of $\sigma =2\;\times \;10^{-2}\;\Omega m^{2}$. This gives an anisotropy ratio of 0.100, showing mixed conduction.

**Figure 22:**
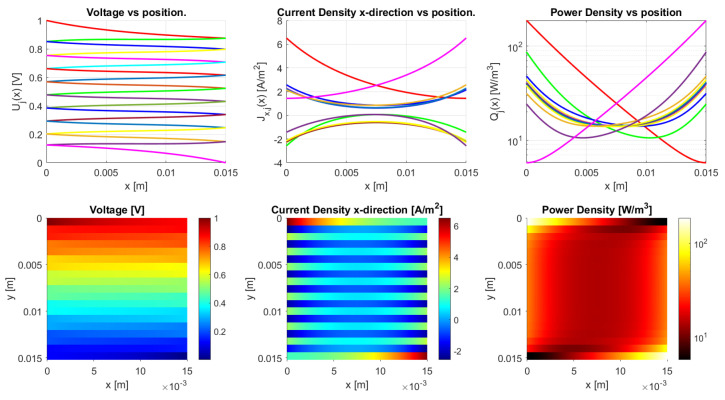
The model results for a meandering sample with the parameters from Table 2 and an inter-traxel resistance of $\sigma =2\;\times \;10^{-1}\;\Omega m^{2}$. This gives an anisotropy ratio of 0.0111, showing traxel conduction.

Bulk conduction is shown in Figure 20 with an anisotropy ratio of $\Gamma_{\mathrm{DC}}=0.918$, where the bulk electrical properties are dominant for the conduction. The voltage drops isotropically over the sample and the current density also spreads evenly over the bulk. The power density is very inhomogeneously distributed, with high peaks that are close to the contacts and deep valleys in the opposite corners. In the case of mixed conduction, the bulk and inter-traxel properties are both of importance for the conduction, as shown in Figure 21 with an anisotropy ratio of $\Gamma_{\mathrm{DC}}=0.1$. Traxel conduction is shown in Figure 22 with an anisotropy ratio of $\Gamma_{\mathrm{DC}}=0.0111$, where the inter-traxel electrical properties determine the conduction. The voltage drop follows the meandering traxels, and the current density is almost constant in the traxels. The power density is spread relatively homogeneously. The power density in the bulk case corresponds to the inhomogeneous heating of samples for bulk conduction and the power density in the traxel case corresponds to the homogeneous heating of samples in [42]. It has to be noted that, in the open-ended case, traxel conduction can be seen as traxels with a constant voltage. The current density does not follow the traxels as for the meandering case.

Accordingly, the value of the anisotropy ratio gives an indication for the type of electrical conduction in a sample, where a very low value indicates highly anisotropic conduction. However, just the value of the anisotropy ratio is not sufficient for predicting the electrical conduction. One also needs to know the number of traxels, the aspect ratio, and the presence of meandering ends or open-ends.

#### 4.2.3. Aspect Ratio

**Figure 23:**
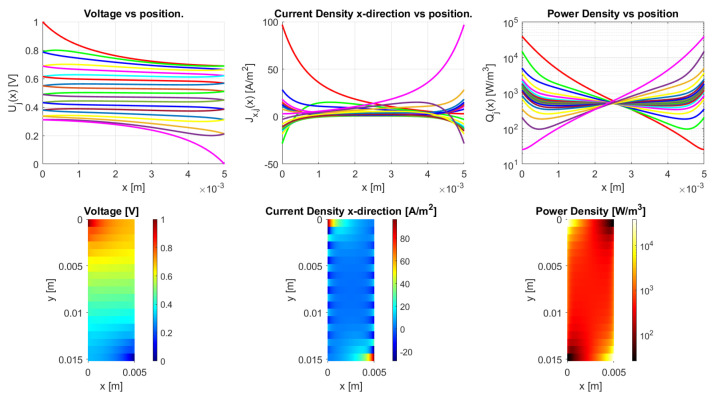
The model results for a meandering thick structure with with traxels of 5 mm long and an aspect ratio of 3.04. The anisotropy ratio is taken to be Γ≈1 by taking $\sigma =2\;\times \;10^{-8}\;\Omega m^{2}$. The voltage drop is mainly from top to bottom.

**Figure 24:**
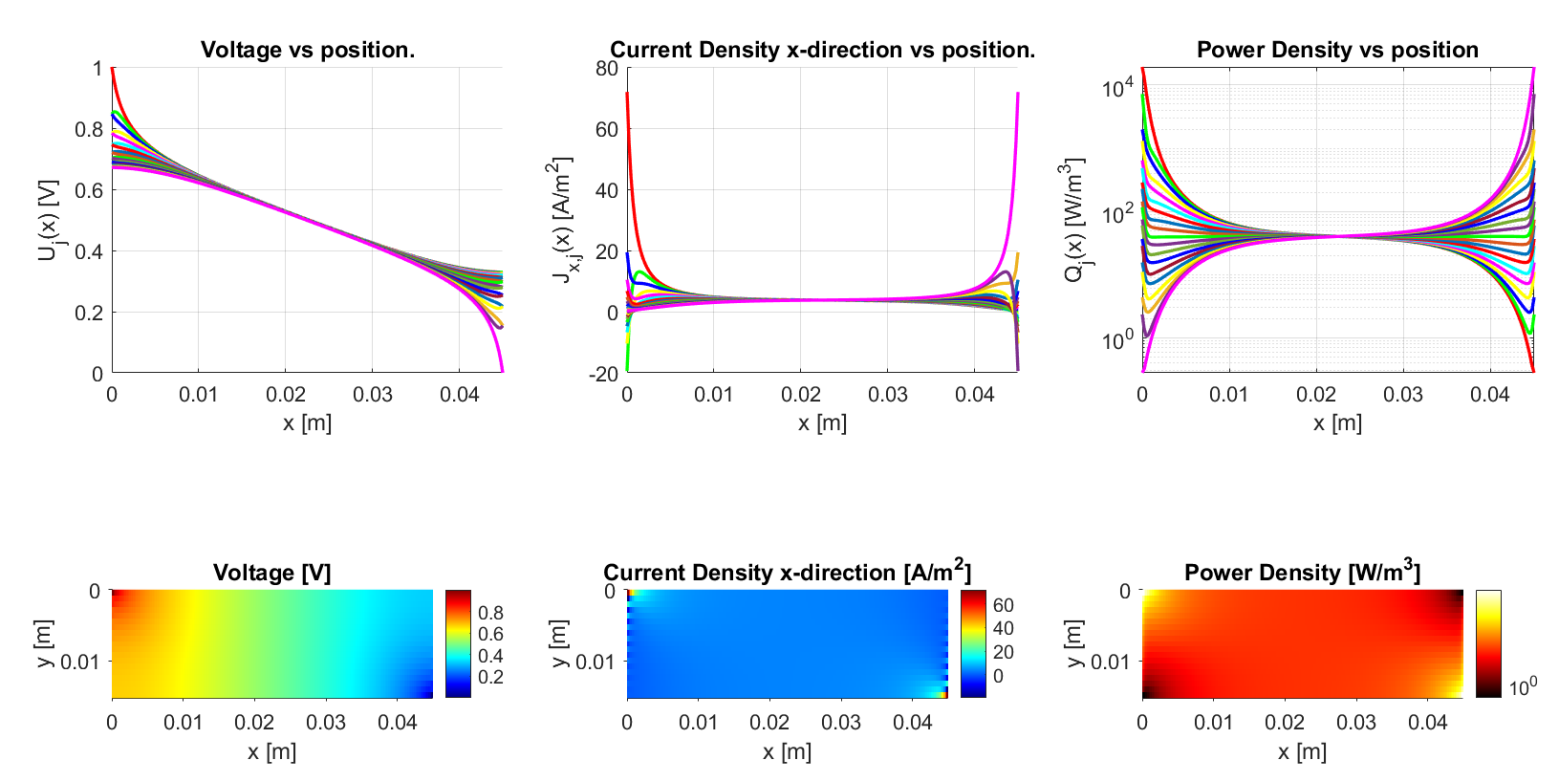
Model results for a meandering, long, slender structure with traxels of 45 mm long and an aspect ratio of 0.338. The anisotropy ratio is taken to be Γ≈1 by taking $\sigma =2\;\times \;10^{-8}\;\Omega m^{2}$. The voltage drop is mainly from left to right.

The aspect ratio strongly determines the ratio between resistance in the vertical and horizontal direction, as the resistance ratio already indicates in Equation (45). When considering a very thick sample (Figure 23) or a long, slender sample, as in Figure 24, they have a different voltage distribution (a voltage drop from top to bottom and from left to right, respectively). This can be more clearly recognized in the voltage versus position graphs, where, for a high aspect ratio, the voltage mainly drops between traxels, as in Figure 23, and, for low aspect ratios, the voltage mainly drops within traxels, as seen in Figure 24.

#### 4.2.4. Number of Traxels

The number of traxels is an important parameter, since it determines the total path length in the case of traxel conduction, and it determines the number of contacts in the sample. The number of traxels becomes important for anisotropy ratios that are close to 1, and for the meandering case in general.

**Figure 25:**
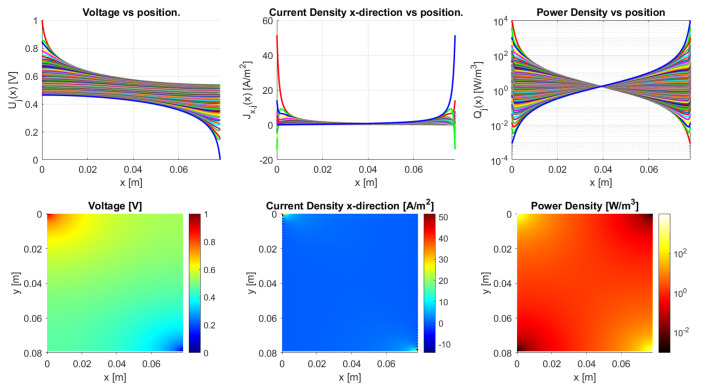
The model converges to a solution of the Laplacian for homogeneous anisotropic materials in the case of large numbers of traxels, in this case 99 traxels.

Wth a large number of traxels and small width, the model converges to the Laplacian for homogeneous anisotropic materials, as described in Section 2.3.2. Such a solution is shown in Figure 25. The discrete nature of the traxels becomes insignificant, as opposed to, for example, Figure 10, where steps in the voltage between traxels can be recognized. The effect of the meandering ends on the resistance becomes insignificant for anisotropy ratios that are close to 1 and large numbers of traxels, which can also be seen from the resistance approximation in Equation (59).

#### 4.2.5. Total Resistance and Effect of Meandering and Open-Ends

**Figure 26:**
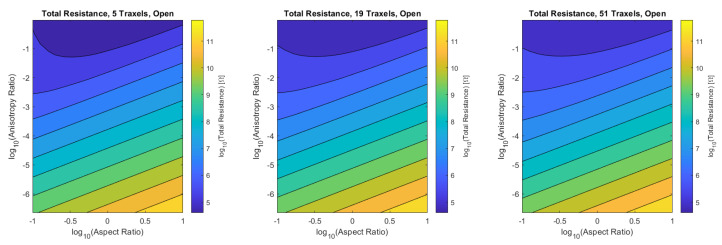
The total resistance for the open-ended model as a function of aspect ratio and anisotropy ratio for 5, 19, and 51 traxels. The resistance is approximately the same for the different numbers of traxels.

**Figure 27:**
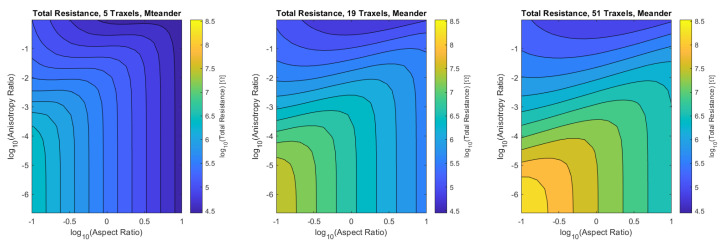
The total resistance for the meandering model as a function of aspect ratio and anisotropy ratio for 5, 19 and 51 traxels. The resistance shows the same qualitative behavior for the different numbers of traxels with a shift in resistance.

Figure 26 and Figure 27 show the total resistance as a function of the dimensionless numbers, where the plots are more general versions of the plots shown in Figure 15 fixed at the aspect ratio AR=1.013 and Figure 14 fixed at the anisotropy ratio Γ=0.528.

For the open-ended case, the total resistance has a minimum around aspect ratios of 1 for anisotropy ratios close to 1. For smaller anisotropy ratios, the total resistance is linear on a logarithmic scale with both the aspect ratio and anisotropy ratio. Under the condition of large aspect ratios and small anisotropy ratios: $\frac{AR^{2}}{\Gamma }\gg 1$, Equation (56) becomes:

(60) $$R_{\mathrm{open}}{|}_{N\gg 1,\frac{AR^{2}}{\Gamma }\gg 1}\approx \frac{\rho }{H}\frac{AR}{\Gamma }$$

Therefore, the relation for a constant total resistance value $R_{\mathrm{const}.}$ in that range is given by $AR\approx R_{\mathrm{const}.}\frac{H}{\rho }\Gamma$, which gives linear results on a logarithmic scale.

The total resistance of the meandering case can be interpreted as the open-ended case with the traxel conduction resistance in parallel, since the meandering ends provide the additional traxel conduction pathway. More traxel conduction will occur if the resistance for bulk conduction becomes larger. This explains the constant resistance for low anisotropy ratios at a fixed aspect ratio in Figure 27, where the resistance converges to the traxel conduction resistance. Traxel conduction is also more prominent for large aspect ratios, since the full traxel conduction path is relatively short in that case. On the other hand, for anisotropy ratios that are close to 1, all cases converge to pure bulk resistance and the effect of meandering ends becomes smaller. In the case of small aspect ratios, the traxel conduction pathway becomes very long and, therefore, bulk conduction becomes more prominent (and the total resistance for the open-ended and meandering case are closer together).

#### 4.2.6. Multiple Inputs

**Figure 28:**
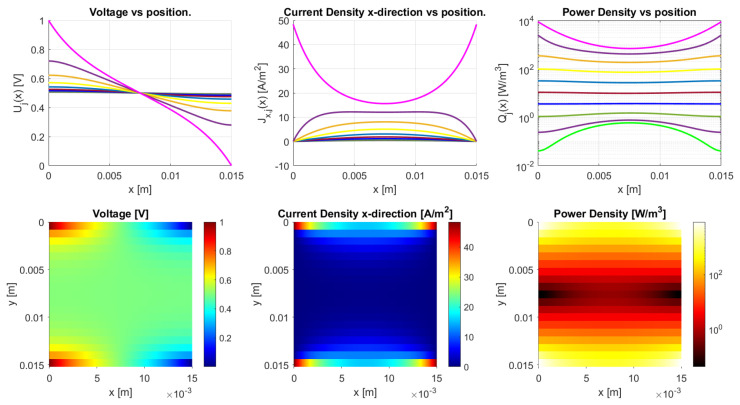
Connecting multiple inputs and outputs is possible, like connecting all corners. In this specific example, open-ended traxels are used with an inter-traxel resistance of $\sigma =2\;\times \;10^{-2}\;\Omega m^{2}$ combined with the other parameters from Table 2, providing an anisotropy ratio of 0.1007.

In the model, it is also possible to connect multiple inputs and outputs. In Figure 28, one can find a geometry with open-ends, where, on the left hand side, the first and last traxel are connected to the input voltage and, on the right hand side, the first and last traxel are connected to output voltage. This can, for example, represent a sample of several layers, where the connections are made on the top and bottom over the entire depth. In this case, it becomes clear that the current mainly runs through the top and bottom layer and barely through the center layers. In this way, multiple inputs and outputs can be used to design spatial voltage dividers or, in the case of ac signals, even spatial filters.

#### 4.2.7. Frequency-Dependent Behavior

**Figure 29:**
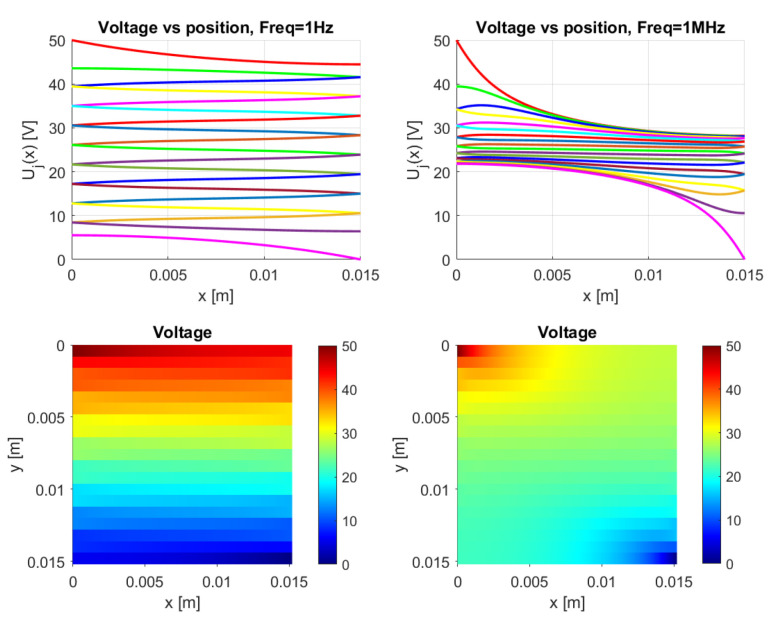
A demonstration of the ac model, showing the voltage plots. Traxel conduction occurs at 1 Hz and bulk conduction occurs at 1 MHz, while using the parameters $C_{0}=2.8\;\times \;10^{-5}\;F\;m^{-2}$, $\epsilon_{r}=5$, ρ=2.8 Ωm, and $\sigma =0.2\;\Omega m^{2}$. According to this model, the frequency-dependence can significantly influence the electrical conduction through 3D-prints.

Section 2 presents the model for frequency-dependent behavior. This frequency-dependence is also implemented in Matlab; however, it has not yet been verified and validated properly. As a first demonstration, Figure 29 presents voltage plots for a frequency of 1 Hz and 1 MHz. The calculation uses a contact capacitance per area of $C_{0}=2.8\;\times \;10^{-5}\;F\;m^{-2}$, a relative permittivity of $\epsilon_{r}=5$, a resistivity of ρ=2.8 Ωm, and an exaggerated contact resistivity of $\sigma =0.2\;\Omega m^{2}$. These values clearly demonstrate traxel conduction at a frequency of 1 Hz, corresponding to Equation (47) with dominant contact resistance, and bulk conduction at a frequency of 1 MHz, which corresponds to Equation (49) with dominant contact capacitance. These results indicate the possibility of using the frequency-dependence to influence the electrical conduction through 3D-prints, where the impedance displays RC-behavior.

### 4.3. Sensor Application

**Figure 30:**
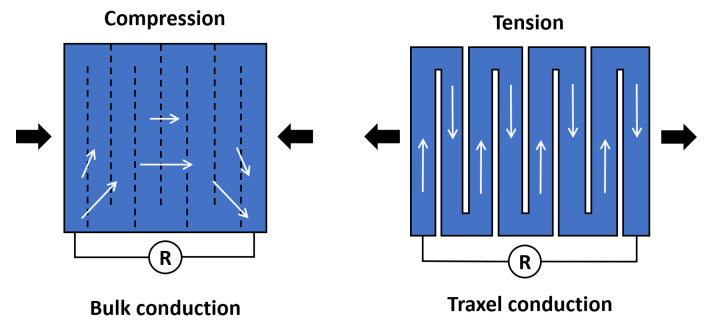
The principle of constriction-resistance strain sensor. The sensing principle makes use of the large resistance change going from bulk conduction to traxel conduction upon straining of the gaps between the traxels. The white arrows indicate the current flow through the sensor, whereas the black arrows indicate the force applied on the sensor.

The model is applied to analyze the performance of so-called constriction-resistive strain sensors [47]; this type of sensor is very similar to the so-called strain-mediated contact in anisotropically resistive structures [48]. Constriction-resistive strain sensors use a meander, which, upon straining, is pulled apart or pushed together, as shown in Figure 30. This changes the inter-traxel resistance (the constriction resistance) upon which the total resistance changes significantly, since the conduction changes from bulk conduction to traxel conduction. This concept is also referred to as resistive path adjustment, where it is used for a 3D-printed sensor that measures the compressive load [49]. The constriction-resistive strain sensors formulated by Mousavi et al., are used as a showcase for the model, since the model can describe it properly with the meanders and contact resistance. The model still lacks the relation between strain and contact resistance; however, this is not required for showing the concept. The values used to study this concept are based on [47], and they are shown in Table 4. The sensor geometry is also derived from the research of Mousavi et al., using electrical leads in opposite corners.

**Figure 31:**
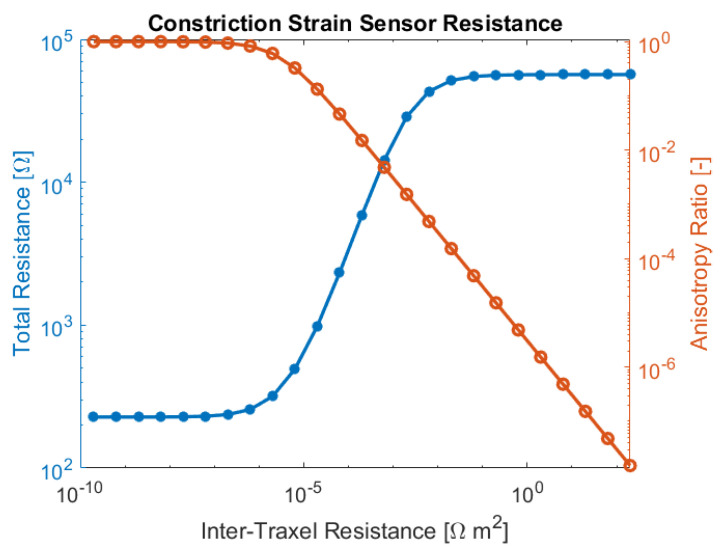
The modelled resistance versus inter-traxel resistance for a constriction-resistive strain sensor, showing a large difference between the lowest and highest resistance. For very low inter-traxel resistance values, the anisotropy ratio is almost 1 and the total resistance stays the same. For very large inter-traxel resistance values, the anisotropy ratio becomes small and, eventually, the total resistance levels out when approaching pure traxel conduction.

**Figure 32:**
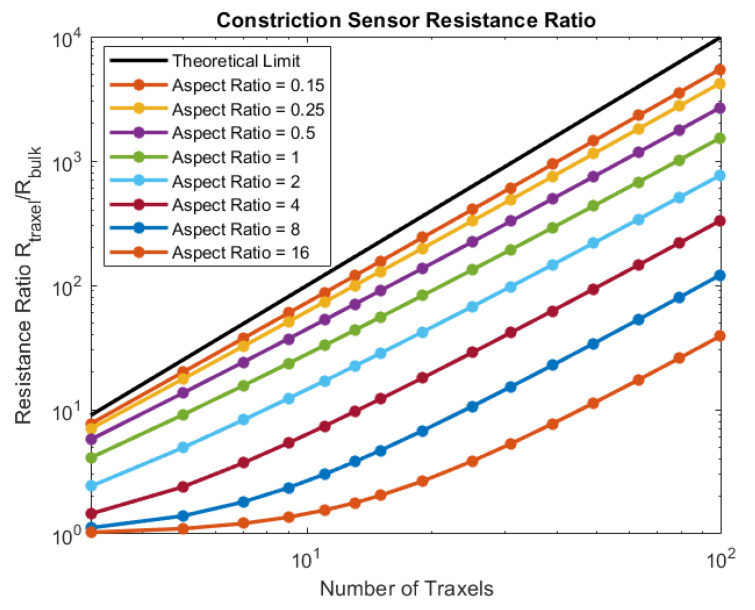
The modeled traxel resistance to bulk resistance ratio as a function of the number of traxels (e.g., the ratio between the maximum and minimum total resistance in Figure 31), for several aspect ratios. Hence, increasing the number of traxels and decreasing the aspect ratio will increase the traxel/bulk resistance ratio of the sensor. In theory, the ratio is limited by the number of traxels, as given in Equation (62).

In Section 4.2.2, different inter-traxel resistance values are used in the model, in order to show the possible effects. The results can be seen in Figure 20, Figure 21 and Figure 22. The voltage and current in the lowest inter-traxel resistance case clearly show bulk conduction, where the traxels barely play a part in the conduction, as seen in Figure 20. For the highest inter-traxel resistance, the voltage drop and current clearly show traxel conduction by following the meandering of the traxels, as in Figure 22. Constriction-resistance strain sensors are based on the change in resistance upon changing conduction mode. A plot of the total resistance as a function of the inter-traxel resistance can be found in Figure 31. It is important to note that the anisotropy ratio keeps going down with an increase of the inter-traxel resistance, whereas the effect on the total resistance reaches a limit. Hence, decreasing the anisotropy ratio only has an effect on the total resistance up to a certain limit, where there is mainly bulk conduction. The large difference between resistance values is indicative of the large resistance differences that were achieved in the literature [47,48]. Besides the inter-traxel resistance, the number of traxels is also an important parameter for this type of sensor, since the amount of contacts scales with the number of traxels. In Figure 32, the ratio between the resistance for bulk and traxel conduction is presented as function of the numbers of traxels for several aspect ratios. It becomes clear that higher numbers of traxels and low aspect ratios provide a larger resistance ratio for this type of sensors. This also becomes clear when using the resistance approximation to roughly predict the traxel to bulk resistance ratio:

(61) $$\mathrm{Ratio}=\frac{R_{\mathrm{traxel}}}{R_{\mathrm{bulk}}}\approx \frac{\frac{\rho }{H}\frac{N^{2}}{AR}}{\frac{\rho }{H}\left(\frac{1}{AR}+AR\right)}=\frac{N^{2}}{1+AR^{2}}$$

This ratio has a theoretical upper limit for very low anisotropy ratios:

(62) $$\lim_{AR\to 0}\mathrm{Ratio}=N^{2}$$

Accordingly, in the limit to small aspect ratios, the traxel to bulk resistance ratio is solely determined by the number of traxels. Indeed, these findings indicate that, for a high traxel to bulk resistance ratio, one should have a low aspect ratio and a large number of traxels.

It has been shown that the model is suited for modeling this type of sensors. Additionally, any type of meandering or sheet-like sensor that depends on inter-traxel impedance can be modeled (e.g. humidity sensors or other types of sensors where a change of electrical conductivity or permittivity of the surroundings has to be measured). In case the relation with the model parameters is known, the sensor performance can be predicted (so, the relation between the physical phenomenon, like temperature or strain, with the model parameters, like resistivity and inter-traxel resistance). Besides sensors, other types of conductive structures can also be studied with the ac model. For example, the network frequency-dependent behavior can be used in the future to print physical filters. The ability of the RC-properties in combination with arbitrary inputs and outputs allows tuning filter properties and embedding them in structures. As another example, the meso-structure with resistive inter-traxel contacts created through FDM has the properties of a metamaterial. Similar anisotropic layered metamaterials are used for the concentrating or cloaking of heat [50,51]. We propose that the infill in 3D-printed conductors can be used to create these structures in the future.

## 5. Discussion

**Figure 33:**
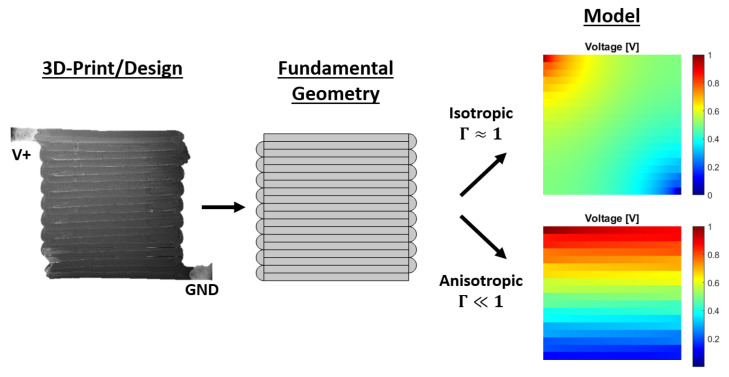
The model can be used to calculate the conduction in 3D-prints from the sample geometry and material properties, over the whole range of conduction behavior from isotropic to anisotropic.

3D-printing conductive structures is gaining attention, especially in the field of 3D-printed sensors. However, the printing process introduces anisotropic electrical properties due to the infill and bonding conditions. Insights in the electrical conduction due to the anisotropic electrical properties are limited in the literature so far. This research focuses on modeling the electrical conduction analytically through a phenomenological model. The electrical properties are described as an electrical network with bulk and contact properties in and between neighbouring printed track-elements, or traxels. The model studies both open-ended and meandering traxels through the boundary conditions. The model equations are solved as an eigenvalue problem, yielding the voltage, current density, and power dissipation density for every traxel. As illustrated in Figure 33, the model can be used to calculate the conduction in 3D-prints over the whole range of conduction behavior from isotropic to anisotropic. The analytical model is verified with lumped resistance approximations and Finite Element Method simulations. Three dimensionless numbers are introduced for the verification and analysis: the anisotropy ratio, the aspect ratio, and the number of traxels. The model results are in correspondence with the FEM simulations, except for cases with anisotropy ratios that are close to 1 (so insignificant inter-traxel resistance), for low numbers of traxels, and, specifically in the meandering case, for large aspect ratios. These errors exist due to limitations of the model with 2D-conduction inside traxels and due to neglecting of the resistance of the meandering ends.

The results show that the dimensionless numbers can be used to analyze the type of conduction and the resulting total resistance. Anisotropy ratios that are close to 1 give bulk or isotropic conduction properties. For low aspect ratios, the effect of both inter-traxel resistance and meanders becomes smaller, since conduction is mainly in the length direction. On the other hand, for large aspect ratios, the inter-traxel resistance is dominant and traxel conduction already occurs for lower anisotropy ratios. The number of traxels also determines the effect of the inter-traxel resistance, since it directly influences the amount of contacts and the traxel conduction path length. Therefore, for low numbers of traxels, the effect of the inter-traxel resistance and, therefore, traxel conduction, become more prominent. It is shown that the conduction through meandering samples can be regarded as the open-ended bulk conduction case in parallel with traxel conduction. Traxel conduction becomes dominant for meandering samples in situations with high bulk conduction resistance and high inter-traxel resistance. It can be seen that the total resistance for meandering samples converges to the traxel conduction resistance for low anisotropy ratios and large aspect ratios.

The use of the model for sensor applications is demonstrated by means of a constriction-resistance strain sensor design study, showing that it is important for this design to have a large number of traxels and a small aspect ratio. This demonstration shows the importance of models in understanding the operation and performance of sensors and new sensing principles, in particular since the manufacturing method FDM is relatively new and its effects on sensors are not yet fully understood. All in all, the model is verified and analyzed, raising several points of discussion.

The model presented is phenomenological in nature. The exact nature of the contact properties cannot be explained from the model, but the effects on the electrical conduction can be expressed through the model by means of inter-traxel properties. Greater research is needed in order to see what the relation is between effects, like the distribution of carbon black, imperfect fusion, and varying layer thickness and the inter-traxel and inter-layer properties. It will be easier to fabricate samples with different anisotropy ratios in the case where a clear relation between these effects and the printing parameters can be found. Additionally, for sensor applications, the relation between the sensor stimulus and inter-traxel properties still needs to be researched, and information like this would significantly improve the usability of the model.

The model currently treats the inter-traxel properties as homogeneous properties of 3D-prints. However, Tronvoll et al. have already been shown in the literature that voids are not homogeneously distributed over the entire printed sample [52], and Truman at al. have shown that an inhomogeneous distribution of conductive properties can significantly influence the conduction [13]. More research is required to understand how this limits the findings from the model. Furthermore, the model is limited to rectangular geometries and the main merit is understanding the fundamental electrical conduction through simple 3D-prints. However, it is possible to study more complex geometries with, for example, different geometries and infill patterns through FEM simulations. It has to be noted that the FEM simulations still need experimental validation for extreme parameter sets and different geometries. Moreover, the model offers quicker evaluations than FEM simulations. The presented resistance approximation can be used to provide a first indication of conduction for extreme parameter sets.

Future research will focus on implementing both the ac properties and the 3D-model. Besides that, the model will be applied to model and improve different sensor applications, which will help to further validate the model. Other types of applications, such as spatial filters and metamaterials, will also be studied. It is expected that the model has merit for a wider audience, working on other types of applications, like printed electronics and heaters, or other types of resistive composites, such asCFRP and conductive textiles. Furthermore, the model also holds merit for other extrusion-based manufacturing methods similar to FDM, such Direct Ink Writing (DIW), where it has already been shown that DIW can be used to fabricate conductive sensors with anisotropic electrical properties [53,54]. The model is open-access and it can be found in [46].

## Figures and Tables

**Table 1:** A summary of the different boundary conditions, where, for the outer *x*-positions, we have ξ=0∨L.

Condition	Voltage BC	Current BC
Applied voltage	${\hat{U}}_{i}\left(\xi ,\omega \right)={\hat{U}}_{\mathrm{in}/\mathrm{out}}$	-
Open-ended	-	$\frac{\partial {\hat{U}}_{n}\left(\xi ,\omega \right)}{\partial x}=0$
Meandering	${\hat{U}}_{i}\left(\xi ,\omega \right)={\hat{U}}_{i+1}\left(\xi ,\omega \right)$	${\hat{I}}_{i}\left(\xi ,\omega \right)=-{\hat{I}}_{i+1}\left(\xi ,\omega \right)$

**Table 2:** The parameters used for modeling and simulations. Data from [42].

Variable/ Sample, Units	Values
Resistivity ρ, Ωm	2.8
Inter-traxel resistivity σ, $\Omega m^{2}$	$2\;\times \;10^{-3}$
Traxel width *W*, mm	0.8
Traxel length *L*, mm	15
Traxel height *H*, μm	200
Number of traxels *N*, -	19
Anisotropy Number $\Gamma_{\mathrm{DC}}$, -	0.528
Aspect Ratio AR, -	1.013

**Table 3:** Root-mean-square (RMS) errors for the errors in Figure 16 and Figure 17.

Number of Traxels:	5	19	51
RMS-error open-ended:	4.75%	2.51%	1.97%
RMS-error meandering:	24.33%	10.26%	4.70%

**Table 4:** Model parameters used for the constriction-resistive strain sensor calculations, based on the work conducted by Mousavi et al. [47].

Parameter	Values
Resistivity ρ	6.8 mΩm
Inter-traxel resistivity σ	2 × 10^−6^ Ωm${}^{2}$–2 × 10^3^ Ωm${}^{2}$
Traxel width *W*	450 μm
Traxel length *L*	10 mm
Traxel height *H*	200 μm
Gauge Length	35 mm
Infill density	95%
Number of traxels *N*	0.95*35 mm/0.45 mm=74≈75

**Figure A2:**
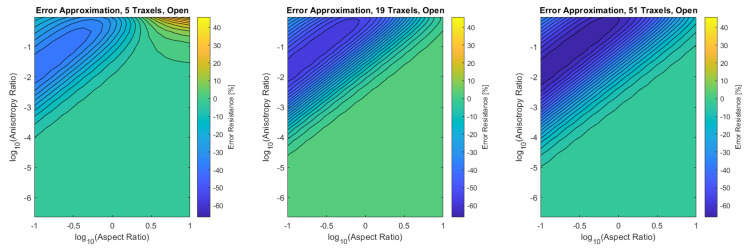
The error of the approximated open-ended resistance compared to the FEM simulations. The RMS-error for the different numbers of traxels is respectively 15.52%, 25.29% and 31.71%.

**Figure A3:**
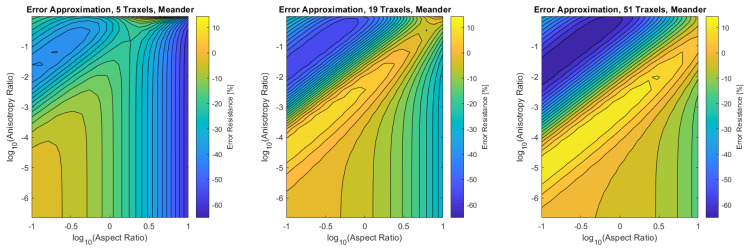
The error of the approximated meandering resistance compared to the FEM simulations. The RMS-error for the different numbers of traxels is respectively 28.39%, 25.25% and 30.59%.

## Supplementary Materials

The Matlab model is available online at [46].

## Abbreviations

The following abbreviations are used in this manuscript:

CB	Carbon Black
CNT	Carbon Nanotubes
CPC	Conductive Polymer Composite
CFRP	Carbon Fiber Reinforced Polymer
FDM	Fused Deposition Modeling
FEM	Finite Element Method
RMS	Root-mean-square
SEM	Scanning Electronc Microscopy
Traxel	Track Element
VCSEM	Voltage Contrast Scanning Electron Miscroscopy

## Appendix A. Equivalent Contact Impedance

In this appendix the total impedance of the equivalent model for the circuit in Figure A1 is derived, combining contact properties and vertical bulk properties. The goal is to obtain a single equivalent impedance as shown in Figure 8. For a single parallel RC-element the impedance becomes:

(A1) $${\hat{Z}}_{\mathrm{RC}-\mathrm{parallel}}=\frac{1}{\frac{1}{R}+j\omega C}=\frac{R}{1+\omega RC}$$

The circuit in Figure A1 contains three of the elements in series (with subscript *b* for bulk properties and *c* for contact properties), for which the impedance can be found by simple addition (because of linearity the two half terms for the bulk properties can be taken as one fraction, which has to be taken into account with the geometry):

(A2) $${\hat{Z}}_{\mathrm{eq}}=\frac{R_{b}}{1+j\omega R_{b}C_{b}}+\frac{R_{c}}{1+j\omega R_{c}C_{c}}$$

Combining both fractions yields the following expression:

(A3) $${\hat{Z}}_{\mathrm{eq}}=\frac{R_{b}+R_{c}+j\omega R_{b}R_{c}C_{c}+j\omega R_{b}R_{c}C_{b}}{\left(1+j\omega R_{b}C_{b}\right)\left(1+j\omega R_{c}C_{c}\right)}$$

The bulk resistance can then be defined as $R_{b}=\frac{\rho W}{H\Delta x}$, the bulk capacitance is described with $C_{b}=\frac{\epsilon_{0}\epsilon_{r}H\Delta x}{W}$, the contact resistance is defined as $R_{c}=\frac{\sigma }{A}=\frac{\sigma }{H\Delta x}$ and finally the contact capacitance can be expressed as $C_{c}=C_{0}H\Delta x$. Substituting these representations yields:

(A4) $${\hat{Z}}_{\mathrm{eq}}=\frac{\frac{\rho W}{H\Delta x}+\frac{\sigma }{H\Delta x}+j\omega \frac{\rho W}{H\Delta x}\frac{\sigma }{H\Delta x}C_{0}H\Delta x+j\omega \frac{\rho W}{H\Delta x}\frac{\sigma }{H\Delta x}\frac{\epsilon_{0}\epsilon_{r}H\Delta x}{W}}{\left(1+j\omega \frac{\rho W}{H\Delta x}\frac{\epsilon_{0}\epsilon_{r}H\Delta x}{W}\right)\left(1+j\omega \frac{\sigma }{H\Delta x}C_{0}H\Delta x\right)}$$

Combining terms and simplifying finally gives the following result:

(A5) $${\hat{Z}}_{\mathrm{eq}}=\frac{1}{H\Delta x}\;\cdot \;\frac{\rho W+\sigma +j\omega \rho \sigma WC_{0}+j\omega \rho \sigma \epsilon_{0}\epsilon_{r}}{1+j\omega \rho \epsilon_{0}\epsilon_{r}+j\omega \sigma C_{0}-\omega^{2}\rho \sigma \epsilon_{0}\epsilon_{r}C_{0}}$$

Equation (A5) is used for the derivation of the model in Section 2.1.2. For the dc implementation the equivalent resistance, as used in the dc model, becomes:

(A6) $$R_{\mathrm{eq}}=\frac{\rho W+\sigma }{H\Delta x}$$

## Appendix B. Extended Model Cases

The presentation of the model in the paper is kept as general and simple as possible, to be able to study the core concepts. For more complex cases several extensions can be made to this model, for example:

- Addition of anisotropic bulk properties, for example for filler particles with large aspect ratios (e.g., carbon nanotubes or even carbon fiber):
  (A7) $$\Gamma =\frac{\left(\frac{\rho_{x}}{1+j\omega \rho_{x}\epsilon_{x}}\right)}{\left(\frac{\rho_{y}}{1+j\omega \rho_{y}\epsilon_{y}}\right)+\left(\frac{\sigma /W}{1+j\omega \sigma C_{0}}\right)}$$
- Extending the model to 3D, by stacking layers of traxels. The system equations can be obtained through a similar derivation as for the 2D-expressions:
  (A8) $$\begin{array}{c} \frac{\partial^{2}{\hat{U}}_{m,n}\left(x,\omega \right)}{\partial x^{2}}+\Gamma_{y}\left(\omega \right)\left({\hat{U}}_{m-1,n}\left(x,\omega \right)-2{\hat{U}}_{m,n}\left(x,\omega \right)+{\hat{U}}_{m+1,n}\left(x,\omega \right)\right)\\ +\Gamma_{z}\left(\omega \right)\left({\hat{U}}_{m,n-1}\left(x,\omega \right)-2{\hat{U}}_{m,n}\left(x,\omega \right)+{\hat{U}}_{m,n+1}\left(x,\omega \right)\right)=0 \end{array}$$
- Including inductive effects and extending impedance properties to next neighbours in the model in case of more dominant capacitive and inductive properties.

## Appendix C. Error Approximated Resistance

The approximated resistance expressions for the open-ended and meandering case is compared to the FEM simulations. The error for the open-ended case is given in Figure A2 and for the meandering case in Figure A3. For both situations the error is the biggest for anisotropy ratios close to 1, since in these cases mainly bulk conduction occurs and for aspect ratios around 1 this cannot be approximated by the horizontal and vertical resistance in series. In case of low anisotropy ratios and low aspect ratios the error becomes significantly lower. For the meandering case the error for large aspect ratios is also significant, since the resistance approximation does not take into account 2D-conduction and the resistance of the meandering ends.
